# Population variability of rhesus macaque (*Macaca mulatta*) *NAT1* gene for arylamine *N-*acetyltransferase 1: Functional effects and comparison with human

**DOI:** 10.1038/s41598-019-47485-x

**Published:** 2019-07-29

**Authors:** Sotiria Boukouvala, Zoi Chasapopoulou, Despina Giannouri, Evanthia Kontomina, Nikolaos Marinakis, Sophia V. Rizou, Ioanna Stefani, Theodora Tsirka, Charlotte Veyssière, Sofia Zaliou, Audrey Sabbagh, Brigitte Crouau-Roy, Giannoulis Fakis

**Affiliations:** 1 Democritus University of Thrace, Department of Molecular Biology and Genetics, Alexandroupolis, Greece; 2 CNRS, Université P. Sabatier, IRD, UMR5174 EDB lab (Laboratoire Evolution & Diversité Biologique), Toulouse, France; 3 UMR 261 MERIT IRD, Faculté de Pharmacie de Paris, Université Paris Descartes, Sorbonne Paris Cité, Paris, France; 4 Present Address: Medicon Hellas S.A., Gerakas Attikis, Greece

**Keywords:** Evolutionary biology, Transferases

## Abstract

Human *NAT1* gene for *N*-acetyltransferase 1 modulates xenobiotic metabolism of arylamine drugs and mutagens. Beyond pharmacogenetics, *NAT1* is also relevant to breast cancer. The population history of human *NAT1* suggests evolution through purifying selection, but it is unclear whether this pattern is evident in other primate lineages where population studies are scarce. We report *NAT1* polymorphism in 25 rhesus macaques (*Macaca mulatta*) and describe the haplotypic and functional characteristics of 12 variants. Seven non-synonymous single nucleotide variations (SNVs) were identified and experimentally demonstrated to compromise enzyme function, mainly through destabilization of NAT1 protein and consequent activity loss. One non-synonymous SNV (c.560G > A, p.Arg187Gln) has also been characterized for human *NAT1* with similar effects. Population haplotypic and functional variability of rhesus *NAT1* was considerably higher than previously reported for its human orthologue, suggesting different environmental pressures in the two lineages. Known functional elements downstream of human *NAT1* were also differentiated in rhesus macaque and other primates. Xenobiotic metabolizing enzymes play roles beyond mere protection from exogenous chemicals. Therefore, any link to disease, particularly carcinogenesis, may be via modulation of xenobiotic mutagenicity or more subtle interference with cell physiology. Comparative analyses add the evolutionary dimension to such investigations, assessing functional conservation/diversification among primates.

## Introduction

The *NAT1* gene (HGNC ID: 7645) for human arylamine *N*-acetyltransferase 1 (UniProt ID: P18440; E.C. 2.3.1.5) was originally characterized for its role in the metabolism of xenobiotics, particularly aromatic amine drugs and mutagens. Apart from its pharmacogenetic significance, *NAT1* has more recently been implicated in cell systems relevant to carcinogenesis^1–3^. The human *NAT1* gene, and its mouse orthologue *Nat2*, begin their expression during preimplantation development^4–6^ and remain transcriptionally active in most embryonic and adult tissues^4,6–11^. Both orthologues are controlled by a conserved Sp1-type housekeeping promoter, adjacent to an upstream non-coding exon^11–13^, and are potentially subject to epigenetic regulation^14,15^. Human *NAT1* is also controlled by a second, more tissue-specific promoter^16^, and regulation has been shown to implicate steroid hormones^17–20^, micro-RNAs^21,22^, protein polyubiquitinylation^23,24^, as well as acetylation of the enzyme and allosteric modulation by adenosine triphosphate^25,26^. Human NAT1 and rodent NAT2 proteins have been demonstrated to acetylate the same arylamine substrates^27^, including p-aminobenzoylglutamate (pABGlu), a catabolic derivative of folate^4,27–30^. *Nat2* knockout mice are apparently healthy, but unable to acetylate pABGlu^31,32^. By contrast, transgenic overexpression of human *NAT1* in mice is not tolerated^33^. Recent studies propose that human NAT1 and rodent NAT2 may catalyze the folate-dependent hydrolysis of acetyl coenzyme A (CoA) in the absence of arylamine^34,35^, but a mechanism is not yet firmly established^36^. Other possible roles of human NAT1, currently under investigation, are relevant to fatty acid biosynthesis^37^, methionine salvage^38^, regulation of reactive oxygen species^39^ and mitochondrial bioenergetics^40^.

A strong association is emerging between human *NAT1* overexpression and cancer, particularly estrogen/progesterone receptor positive tumours of both female and male breast (see earlier reviews^2,3,41^ and more recent literature^42–48^). The mechanism underlying the function and regulation of *NAT1* in carcinogenesis is extensively investigated, as the gene is considered to be of prognostic and potentially therapeutic relevance^2,21,22,49–54^. Synthetic NAT1-selective inhibitors are also available^55–57^.

The evolutionary history of human *NAT1* gene indicates a dominant role of purifying selection, consistent with the presumed endogenous function of NAT1 enzyme^58,59^. However, a recent study suggests that the evolutionary pattern may be different for the *NAT1* orthologues of other primate species^60^. We have recently analysed the *NAT1* sequences of 35 non-human primate species and performed recombinant expression and comparative enzymatic analysis for ten of them, elucidating their genetic evolution and demonstrating functional variability across different phylogenetic lineages^61–63^. Here, we describe a series of *NAT1* variants found in a small population sample of rhesus macaques (species *Macaca mulatta*; taxon ID: 9544; UniProt taxon mnemonic: MACMU) and investigate the functional effects of non-synonymous single nucleotide variations (SNVs), drawing parallels with the effects of characterized *NAT1* SNVs in humans.

## Results

### Variability in the rhesus macaque (MACMU)*NAT1* gene

The intronless coding region of (MACMU)*NAT1* gene was sequenced for 25 rhesus macaque individuals. A total of 12 polymorphic sites were identified, of which 5 represented synonymous and 7 non-synonymous nucleotide substitutions relative to reference allele (MACMU)NAT1*1 (Nucleotide ID: KU640969.1)^61^. The determined genotypes (Supplementary Table S1) corresponded to 12 haplotypes (Table 1), which were assigned allelic symbols (MACMU)NAT1*2 – (MACMU)NAT1*12 (Nucleotide IDs: KU640986 *–* KU640996). Allele (MACMU)NAT1*1 showed highest identity (96.45%) to (HUMAN)NAT1*4 reference allele (Nucleotide ID: AJ307007.1) and was also most frequently encountered in the rhesus macaque sample screened, representing 34% of the 50 haplotypes analysed in total. Other frequently found haplotypes were (MACMU)NAT1*3 (18%) and (MACMU)NAT1*5 (20%), while the remaining haplotypes were less common (2–6%). With the exception of (MACMU)NAT1*3 and *8, all haplotypes carried more than one nucleotide variation compared to (MACMU)NAT1*1. Haplotypes (MACMU)NAT1*3, *5, *8, *9 and *12 contained only synonymous substitutions. Haplotypes (MACMU)NAT1*2, *4, *6, *7 and *11 contained one non-synonymous, together with one or two synonymous substitutions. (MACMU)NAT1*10 allele carried three non-synonymous and one synonymous substitution (Table 1).

**Table 1:** (MACMU)*NAT1* haplotypes and corresponding amino acid changes^a,b^.

(MACMU)*NAT1* allele	Nucleotide variation												Ν	Nucleotide ID
	c.15	c.152	c.177	c.244	c.267	c.321	c.343	c.463	c.493	c.523	c.540	c.560		
ΝΑΤ1*1 (Reference)	A	**G**	C	**A**	**G**	C	**G**	**G**	A	**T**	C	**G**	17	KU640969
ΝΑΤ1*2	.	.	.	**G**	.	T	.	.	.	.	T	.	1	KU640986
ΝΑΤ1*3	.	.	.	.	.	T	.	.	.	.	.	.	9	KU640987
ΝΑΤ1*4	.	**C**	.	.	.	T	.	.	.	.	.	.	1	KU640988
ΝΑΤ1*5	.	.	.	.	.	T	.	.	.	.	T	.	10	KU640989
ΝΑΤ1*6	.	.	.	.	.	T	.	.	.	**C**	T	.	1	KU640990
ΝΑΤ1*7	.	.	.	.	.	T	.	.	.	.	.	**A**	3	KU640991
ΝΑΤ1*8	G	.	.	.	.	.	.	.	.	.	.	.	2	KU640992
ΝΑΤ1*9	.	.	.	.	.	T	.	.	C	.	.	.	3	KU640993
ΝΑΤ1*10	.	.	.	.	**C**	T	**T**	**C**	.	.	.	.	1	KU640994
ΝΑΤ1*11	.	.	.	.	.	T	**T**	.	.	.	.	.	1	KU640995
ΝΑΤ1*12	.	.	T	.	.	T	.	.	.	.	.	.	1	KU640996
(MACMU)NAT1 protein	Amino acid variation													
	p.5	**p**.**51**	p.59	**p**.**82**	**p**.**89**	p.107	**p**.**115**	**p**.**155**	p.165	**p**.**175**	p.180	**p**.**187**		
Reference	Ala	**Gly**	Val	**Met**	**Leu**	His	**Asp**	**Glu**	Arg	**Phe**	Leu	**Arg**		
Polymorphic	Ala	**Ala**	Val	**Val**	**Phe**	His	**Tyr**	**Gln**	Arg	**Leu**	Leu	**Gln**		

^a^Polymorphic sites are shown, numbered relative to either the nucleotide sequence of (MACMU)*NAT1* intronless coding region (top part) or the deduced amino acid sequence of (MACMU)NAT1 protein (bottom part), where position c.1 is the adenosine of the translation initiation codon (ATG) and p.1 is the first amino acid (Met) of the polypeptide chain, respectively. Non-synonymous substitutions are shown in bold. N is the number of times each haplotype was encountered in the sample studied (50 chromosomes analysed in total). Assigned allelic symbols and GenBank accession numbers (Nucleotide IDs) are provided for each haplotype.

^b^Estimated haplotype diversity (Hd) in the population was 0.817.

Of the SNVs found (Tables 1 and S1), synonymous substitution c.321C > T was most frequent (52.5%) and altered the codon for key catalytic residue p.His107^64^, which however remained conserved. Synonymous SNV c.540C > T was also common (20.3%), while other synonymous SNVs showed lower frequencies (up to 5%). Of the non-synonymous SNVs (Tables 1 and S1), c.152G > C (p.Gly51Ala), c.244A > G (p.Met82Val), c.267G > C (p.Leu89Phe), c.463G > C (p.Glu155Gln) and c.523T > C (p.Phe175Leu) were encountered only once in the rhesus macaque sample. Non-synonymous substitution of amino acid residue p.Gly51 (c.152G > T, p.Gly51Val; rs72466457:G > T) has been reported previously for the *6 *M* allele of (HUMAN)*NAT2* gene (the 87.4% conserved *NAT1* paralogue in human) and has been predicted to potentially affect substrate binding^65^. Substitution c.343G > T (p.Asp115Tyr) was found in two heterozygous rhesus macaque individuals carrying haplotypes NAT1*10 and *11. The most frequent (5%) non-synonymous SNV in the rhesus macaque sample was c.560G > A (p.Arg187Gln), found in three heterozygous individuals (Tables 1 and S1). This exact SNV (rs4986782:G > A) has been reported for (HUMAN)NAT1*14, with 1000 Genomes Project phase 3 global minor allele frequency (MAF) of 0.6%, and has important functional consequences. The same codon is also subject to another mutation (c.559C > T, p.Arg187Ter; rs5030839:C > T with global MAF of 0.3%) in the (HUMAN)NAT1*15 allele, causing premature termination of the polypeptide chain^66–71^.

**Figure 1:**
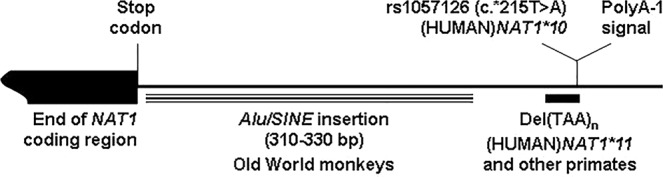
Genomic variability downstream of *NAT1* coding region in primates. A typical *Alu/SINE* element is found about 10 bp after the stop codon of *NAT1* gene in Old World monkeys (11 species, including the rhesus macaque), but is absent in apes (6 species, including human). Moreover, a microsatellite (TAA)_8_ repeat element, spanning one consensus polyadenylation signal (polyA-1) of (HUMAN)*NAT1* gene, is 12–18 bp shorter in all 16 non-human primate genomes analysed, resembling (HUMAN)NAT1*11 (9 bp shorter) and other polymorphic *NAT1* alleles reported in human populations (http://nat.mbg.duth.gr/). Also shown is SNV rs1057126, which affects polyA-1 signal in (HUMAN)NAT1*10 and other reported human alleles. The figure was not drawn to scale. Detailed illustrations of this particular genomic region are provided in Supplementary Figs S1 and S2.

Although non-synonymous SNVs have been reported in the literature for (HUMAN)*NAT1* gene (http://nat.mbg.duth.gr/), the most common polymorphic alleles in human populations are characterized by functional variability in the 3′-untranslated region (3′-UTR). Allele (HUMAN)NAT1*10 is defined by rs1057126:T > A (c.*215T > A) which affects one active polyadenylation (polyA) signal of the gene. Allele (HUMAN)NAT1*11 is defined by a shortage of three TAA repeats in a microsatellite element spanning the same polyA signal. This particular region is highly variable in human populations (http://nat.mbg.duth.gr/), and has been demonstrated to modulate polyA usage of the gene and mRNA translation efficiency^72^. We show here that the rhesus macaque and other non-human primates (both Old World monkeys and apes) also carry a shorter microsatellite stretch at this position, suggesting an expansion of the ancestral wild-type allele specific to the human evolutionary lineage (Figs 1 and S1). Moreover, the genomic distance of this particular microsatellite/polyA region from the stop codon of *NAT1* gene is about 300 bp longer in the rhesus macaque and other Old World monkeys, compared with human and other apes. This difference in length is due to an *Alu/SINE* element which is present immediately downstream of *NAT1* gene in Old World monkeys, but not in apes (Figs 1 and S2). These notable differences between human and other primates suggest substantial evolutionary and functional divergence of the *NAT1* 3′-UTR that merits further investigation, especially given the phenotypic significance attributed to (HUMAN)NAT1*10 and *11 alleles and their investigated association with disease^2,72^.

### Effects of polymorphisms on rhesus macaque (MACMU)NAT1 protein expression

A clone carrying the (MACMU)NAT1*1 reference allele in recombinant expression vector^61^ was used to generate a series of mutagenised constructs, each carrying one of the seven non-synonymous SNVs found in the rhesus macaque sample screened. One additional construct was engineered to carry both c.267G > C (p.Leu89Phe) and c.343G > T (p.Asp115Tyr), as those two SNVs were found together on the same haplotype (Supplementary Table S2).

**Figure 2:**
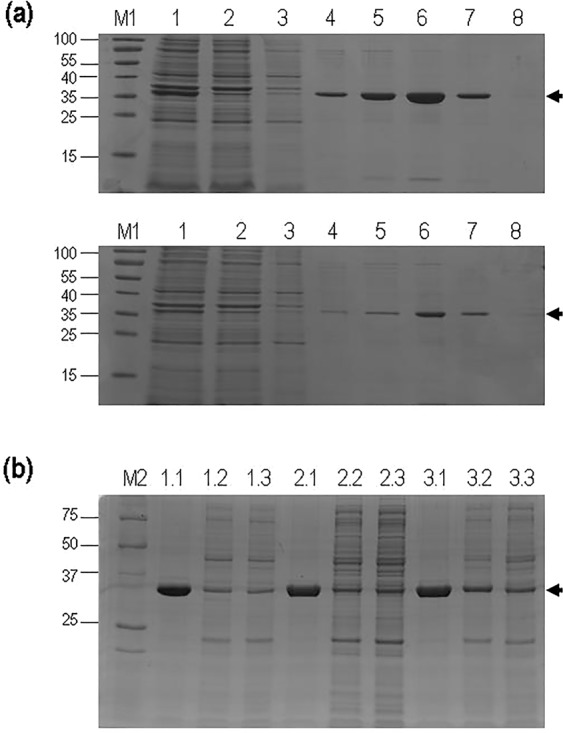
Recombinant (MACMU)NAT1 protein variants. Panel a: Example of the applied protein purification scheme, showing recombinant preparations of reference (MACMU)NAT1_1 (top) and variant (MACMU)NAT1_p.Arg187Gln (bottom) proteins. The two preparations were processed together and in exactly the same way. Both SDS-PAGE gels were loaded with 15 μl of total soluble protein recovered after cell lysis (lane 1), initial flowthrough of the affinity chromatography column (lane 2), as well as the chromatographic fractions eluted with 10, 25, 50, 100, 200 and 250 mM imidazol (lanes 3–8, respectively). Lane M1 is the protein markers (PageRuler Prestained Protein Ladder, Thermo-Fisher). Panel b: SDS-PAGE gel loaded with 10 μl of the 100 mM imidazol fractions of recombinant preparations in the following order: lanes 1.1–1.3, (MACMU)NAT1_1, (MACMU)NAT1_p.Phe175Leu and (MACMU)NAT1_p.Asp115Tyr; lanes 2.1–2.3, (MACMU)NAT1_1, (MACMU)NAT1_p.Met82Val and (MACMU)NAT1_p.Gly51Ala; lanes 3.1–3.3, (MACMU)NAT1_1, (MACMU)NAT1_p.Leu89Phe and (MACMU)NAT1_p.Glu155Gln. The recombinant proteins (reference plus two variants) were processed together as a single set and all three sets were subjected to the same standardized experimental protocol. Lane M2 is the protein marker (Precision Plus Protein Standards, BioRad). In all three gels, the molecular weight (in kDa) of protein markers is shown on the left-hand side. Arrows on the right-hand side indicate bands of recombinant (MACMU)NAT1 proteins. Full-length gels are presented in Expanded Data Supplementary Fig. 1 at the end of the Supplementary Information File.

As previously observed^61^, recombinant expression/purification of (MACMU)NAT1_1 reference protein was always efficient, generating preparations of high yield and purity (Fig. 2). Variant (MACMU)NAT1_p.Arg187Gln also generated sufficient yields of highly pure protein, although at considerably lower levels (by 30% on average) compared with (MACMU)NAT1_1 (Fig. 2). Recombinant expression of all other variants generated poor quality preparations that made recovery of pure proteins virtually impossible, although the expected NAT1 protein band was always visible on gels (Fig. 2). The recombinant preparations of (MACMU)NAT1_1 and (MACMU)NAT1_p.Arg187Gln were in fact differentiated from those of all other variants, throughout the entire expression/purification process (Supplementary Fig. S3). Those results were reproducible upon expression of recombinant (MACMU)NAT1 protein variants multiple (up to five) times and by different (up to three) persons, over an extended period of time. Procedures always incorporated (MACMU)NAT1_1, as reference protein, and followed a well-standardized protocol in our laboratory^61,73,74^ that has been successfully applied to numerous recombinant NAT homologues across a wide taxonomic spectrum of organisms, including the rhesus macaque and other primates. Our observations were consistent with protein aggregation involving misfolded recombinant (MACMU)NAT1 unstable variants, both during expression (in the form of bacterial inclusion bodies), as well as in cell lysates and during purification (in the form of soluble and insoluble complexes with native *Escherichia coli* proteins)^75–77^. Although a variety of approaches are available for overcoming recombinant protein aggregation^75^, optimizing the expression/purification conditions for individual (MACMU)NAT1 variants was beyond the scope of this study, which focused on comparison of SNV effects using a standard procedure widely employed by NAT researchers performing similar work^27,35,64,78–87^. As expression of mammalian proteins in *E*. *coli* could potentially confound the observed functional impact of different SNVs, future investigations of the characterized (MACMU)NAT1 variants could additionally employ eukaryotic cells. However, previous expression of (HUMAN)NAT1 and its variants in standard eukaryotic systems has been demonstrated to generate recombinant protein in very low yield that is barely detectable^70,71^.

**Figure 3:**
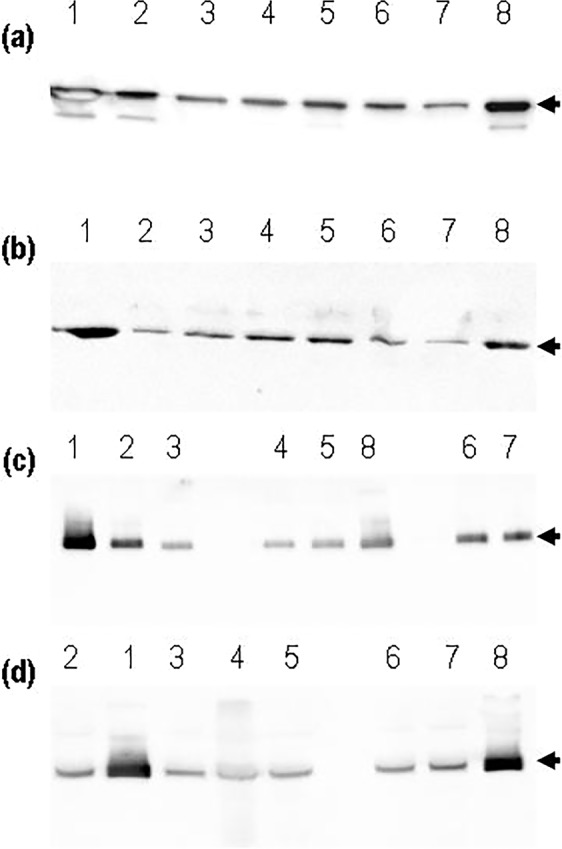
Immunoblot detection of recombinant (MACMU)NAT1 protein variants. Blotted SDS-PAGE gels were loaded with recombinant proteins as follows: (MACMU)NAT1_1 (lane 1), (MACMU)NAT1_p.Phe175Leu (lane 2), (MACMU)NAT1_p.Asp115Tyr (lane 3), (MACMU)NAT1_p.Met82Val (lane 4), (MACMU)NAT1_p.Gly51Ala (lane 5), (MACMU)NAT1_p.Leu89Phe (lane 6), (MACMU)NAT1_p.Glu155Gln (lane 7) and (MACMU)NAT1_p.Arg187Gln (lane 8). Immunodetection took place with antibodies A7058 (**a**), ARP44197 (**b**) and #183 (**c,d**), and the expected (MACMU)NAT1 protein bands are indicated with an arrow. Gels a-c were loaded with 1 μg of protein recovered in the 100 mM imidazol fraction of each recombinant preparation. Gel d was loaded with 6 μl of total soluble protein recovered after cell lysis. Multiple exposures of full-length blots are presented in Expanded Data Supplementary Fig. 1 at the end of the Supplementary Information File.

To verify the presence of recombinant (MACMU)NAT1 protein in preparations of expressed variants, a series of immunoblot analyses were performed, using antibodies specific for the N-terminal, middle or C-terminal part of the polypeptide chain. All preparations were demonstrated to contain recombinant (MACMU)NAT1 protein, both in the soluble fraction of each cell lysate and in the products of subsequent chromatographic purifications (Fig. 3). However, compared with (MACMU)NAT1_1 reference protein, the amount of detected variants was substantially decreased (typically by more than 50%) in recombinant preparations, and this result was consistent across different experiments and with all three antibodies. As expected, (MACMU)NAT1_p.Arg187Gln appeared to be only moderately affected, relative to (MACMU)NAT1_1 reference protein, demonstrating up to 45% reduction in the amount of recombinant protein detected in purified chromatographic fractions. By contrast, the amount of immunodetected (MACMU)NAT1_p.Arg187Gln protein was similar to (MACMU)NAT1_1 reference protein in soluble fractions generated immediately after cell lysis (Fig. 3).

The combination of two amino acid substitutions in the same polypeptide chain had an additive, essentially detrimental, impact on the recombinant expression of (MACMU)NAT1_p.Leu89Phe/p.Asp115Tyr variant. The double mutant was barely detectable by immunoblot analyses, even in freshly prepared cell lysates following recombinant expression (Supplementary Fig. S4).

Overall, these results indicate that (MACMU)NAT1 variants are inherently unstable, when expressed in *E*. *coli*, suggesting that different non-synonymous substitutions may compromise protein folding and solubility. This effect is relatively moderate in the case of (MACMU)NAT1_p.Arg187Gln variant and most pronounced in the case of double mutant (MACMU)NAT1_p.Leu89Phe/p.Asp115Tyr. Bacteria possess complex systems to assess correct folding of expressed proteins and can reject misfolded proteins through proteolysis or the formation of insoluble aggregates^76^. Although application of more sophisticated methods^75^ for detection and monitoring of protein aggregate formation may enable more accurate assessments in the future, the present work demonstrates that the likely impact of characterized non-synonymous SNVs is to compromise the inherent stability of (MACMU)NAT1 protein, as summarized in Table 2. This is in line with previous investigations of (HUMAN)NAT1, demonstrating a similar effect on protein stability for most characterized SNVs, irrespectively of whether the expression was carried out in prokaryotic (*E*. *coli*) or eukaryotic (yeast or mammalian) cells^23,70,71,88^.

**Table 2:** Summary of effects of non-synonymous polymorphisms on (MACMU)NAT1 enzyme function.

Recombinant variant	Observed effect on stability^a^	Observed effect on enzymatic activity^b^	Predicted effect on structure^c^	Predicted effect by PolyPhen-2^c^
(MACMU)NAT1_1	Reference (100%)	Reference (100%)	Reference	Reference
(MACMU)NAT1_p.Gly51Ala	Substantial decrease (40%; 33–51%)	Moderate decrease (55%; 45–69%)	Benign (residue on the surface of the protein molecule)	Benign (score 0.118)
(MACMU)NAT1_p.Met82Val	Substantial decrease (32%; 20–49%)	Moderate decrease (68%; 56–81%)	Benign (residue on the surface of the protein molecule)	Benign (score 0.128)
(MACMU)NAT1_p.Leu89Phe	Substantial decrease (44%; 24–65%)	Substantial decrease (31%; 25–44%)	Damaging (residue close to the catalytic core of the enzyme)	Probably damaging (score 1.000)
(MACMU)NAT1_p.Asp115Tyr	Substantial decrease (32%; 25–39%)	Substantial decrease (18%; 11–26%)	Damaging (residue stabilizes structural conformation important for enzyme function)	Possibly damaging (score 0.845)
(MACMU)NAT1_p.Leu89Phe/p.Asp115Tyr	Barely detectable (<5%)	Residual activity (<5%)	Damaging (combined effect of two compromising changes)	N/A
(MACMU)NAT1_p.Glu155Gln	Substantial decrease (38%; 10–68%)	Marginal/moderate decrease (78%; 44–100%)^b^	Benign (residue on the surface of the protein molecule)	Benign (score 0.009)
(MACMU)NAT1_p.Phe175Leu	Substantial decrease (50%; 11–73%)	Substantial decrease (18%; 15–20%)	Damaging (residue stabilizes structural conformation important for enzyme function)	Possibly damaging (score 0.545)
(MACMU)NAT1_p.Arg187Gln	Marginal/moderate decrease (100%) *vs*. (73%; 56–82%)^a^	Substantial decrease (27%; 20–36%)	Damaging (residue interacts with components important for enzymatic function)	Benign (score 0.444)

^a^The effect of polymorphisms on stability of (MACMU)NAT1 variants was assessed based on observations during recombinant expression/purification processes, the appearence of recombinant proteins on SDS-PAGE gels and immunoblots, as well as on thermal stability assays performed where possible. In parentheses, the average amount and corresponding amount range of immunodetected proteins is provided relative (%) to (MACMU)NAT1_1, and a 50% threshold was used to assess the overall impact of various SNVs on protein stability. The amount of (MACMU)NAT1_p.Arg187Gln protein showed a moderate decrease in the 100 mM imidazol fraction eluted during chromatographic purification, but such decrease was not evident when immunodetection of the same recombinant variant took place in soluble fractions generated immediately after cell lysis.

^b^A 50% enzymatic activity threshold, relative to the (MACMU)NAT1_1 reference protein, was used to assess the effect of each polymorphism as either “moderate” or “substantial”. The average value and corresponding range of relative (%) specific activities measured with PABA, 5AS and PANS are provided in parentheses. Variant (MACMU)NAT1_p.Glu155Gln provided a broad range of activities, depending on which substrate was used, and the overall effect was assessed as “marginal/moderate”.

^c^The predicted effect of each polymorphism on (MACMU)NAT1 protein structure/function was assessed by structural modelling and PolyPhen-2 software.

### Effects of polymorphisms on rhesus macaque (MACMU)NAT1 enzyme activity

**Figure 4:**
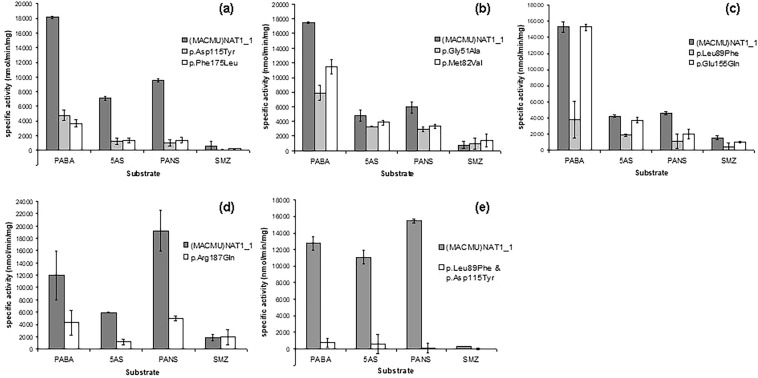
Enzymatic activities of (MACMU)NAT1 variants. Recombinant proteins were processed as separate sets (diagrams **a**–**e**), during preceding expression/purification steps and presented enzymatic activity assays, and all sets were handled according to the same protocol. Each set contained a preparation of (MACMU)NAT1_1 recombinant protein, as reference, plus one or two of its variants. Duplicate reactions were performed with acetyl-CoA as donor substrate and p-aminobenzoic acid (PABA), 5-aminosalicylic acid (5AS), p-anisidine (PANS) and sulphamethazine (SMZ) as acceptor substrates.

The recombinant preparations of (MACMU)NAT1_1 reference protein and its eight variants were assayed for enzymatic activity, using the arylamines p-aminobenzoic acid (PABA), 5-aminosalicylic acid (5AS), p-anisidine (PANS) and sulphamethazine (SMZ) as acetyl-group acceptor substrates. SMZ is a NAT2-selective substrate that was expected to provide only marginal activity with (MACMU)NAT1 protein, unless any of the assayed variants caused a switch in substrate specificity of the isoenzyme, as has been reported previously for the p.Val231Ile polymorphism of (MACMU)NAT2^74,89^. No such effect was demonstrated for any of the (MACMU)NAT1 polymorphisms tested in the present study (Fig. 4).

Because of the apparent instability of (MACMU)NAT1 variants, and the consequently unavoidable variability in the amount and purity of recovered recombinant proteins across different preparations (Table 2), a 50% threshold was used to interpret the effects of polymorphisms on enzyme activity, relative to (MACMU)NAT1_1 reference protein which was included in each set of assays. Enzymatic activity was detected for all variants, when PABA, 5AS or PANS were used as substrates, but was decreased relative to (MACMU)NAT1_1 (Fig. 4). The smallest change was observed for variant (MACMU)NAT1_p.Glu155Gln, which was >90% active relative to (MACMU)NAT1_1, when PABA and 5AS were used. However, a 55% decrease in activity was observed with PANS, suggesting that the effects of the polymorphism could be substrate-specific; PABA and 5AS are more NAT1-selective, compared with PANS which is a substrate for both NAT1 and NAT2 isoenzymes of primates^61^. Most severely compromised was the double mutant (MACMU)NAT1_p.Leu89Phe/p.Asp115Tyr, demonstrating some activity only with PABA. Variants (MACMU)NAT1_p.Gly51Ala and (MACMU)NAT1_p.Met82Val demonstrated moderate (<50%) decrease in enzymatic activity relative to (MACMU)NAT1_1, while the decrease was more substantial (>50%) in the case of variants (MACMU)NAT1_p.Leu89Phe, (MACMU)NAT1_p.Asp115Tyr, (MACMU)NAT1_p.Phe175Leu and (MACMU)NAT1_p.Arg187Gln, as summarized in Table 2.

**Figure 5:**
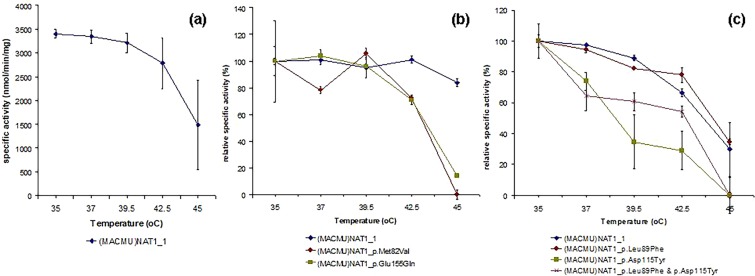
Thermal stability of (MACMU)NAT1 variants. Examples of enzymatic assays used to assess thermal stability of recombinant (MACMU)NAT1 variants. Sets of experiments, testing (MACMU)NAT1_1 reference protein alongside different variants, involved thermal stress for 10 min across a temperature range of 35–45 °C, followed by enzymatic activity assays with PABA in duplicate reactions. The decline in specific activity of (MACMU)NAT1_1 is shown in (**a**), based on four independent sets of experiments with different recombinant preparations. In b and c, the enzyme specific activity measured for each recombinant protein at 35 °C was considered as 100% and was used to record the decline in activity across the temperature gradient. Note that the actual specific activities measured for different variants were considerably lower compared with (MACMU)NAT1_1 protein, because of their inherent instability and functional impairement even without thermal stress.

Initial attempts to assess the impact of substitutions on thermal stability of (MACMU)NAT1 protein, through measurement of enzymatic activity after exposure of recombinant preparations to temperatures from 35 to 45 °C, were complicated by the fact that most variants demonstrated very low enzymatic activities even before thermal stress. In those cases where the results were assessed as reliable, thermal stability of variants appeared to decline at different rates (Fig. 5). Loss of activity was marginal for (MACMU)NAT1_1 reference protein at temperatures close to its reported denaturation midpoint temperature (36.5 ± 1.2 °C)^61^, which is also similar to the body temperature (37.3 °C) of the animal (Animal Ageing and Longevity Database, http://genomics.senescence.info/species/)^90^, but became more evident above 39.5 °C. Variant (MACMU)NAT1_p.Leu89Phe appeared rather resilient to thermal stress, losing activity at similar rate as the reference protein, despite its demonstrated instability during expression/purification. Other variants were more sensitive to the effects of thermal stress (Fig. 5). To overcome the aforementioned limitations, we also tried differential scanning fluorimetry, a well-standardized method in our laboratory^61,73,74^, to assess thermal stability of (MACMU)NAT1 variants. However, with the exception of the reference protein, this approach was hampered by the insufficient purity of recombinant preparations. In the future, it may be useful to try alternative thermal denaturation assays, described in the literature^91^.

Combined, the observations of this and the previous section indicate inherent instability of (MACMU)NAT1 variants as the likely cause for reduced enzymatic activity. However, an effect on catalytic function may not be ruled out, particularly for variants with polymorphisms p.Asp115Tyr and p.Phe175Leu that demonstrated a decrease in enzymatic activity that was considerably more substantial relative to the decrease in the amount of recovered recombinant protein (Table 2). Impairement of catalytic function is also likely to be the main cause for the observed decrease in the enzymatic activity of variant (MACMU)NAT1_p.Arg187Gln, which appeared relatively stable compared with other variants in this study. Polymorphism p.Arg187Gln has also been demonstrated to cause decrease in activity of (HUMAN)NAT1 homologue, both when using human biological material and after recombinant expression in bacterial, yeast or mammalian cell systems^68–71,92–94^. As in the present study for (MACMU)NAT1, the effect of p.Arg187Gln on (HUMAN)NAT1 appeared to be in the form of moderate decrease in protein stability, combined with impairement of enzymatic function^68,70,71^. More sophisticated kinetic analyses with a range of substrates may help elucidate the exact mechanism underlying functional impairement of each characterized (MACMU)NAT1 variant, at least in those cases where stability and activity are sufficient to allow reliable enzymatic assays.

### Structural effects of rhesus macaque (MACMU)NAT1 polymorphisms

**Figure 6:**
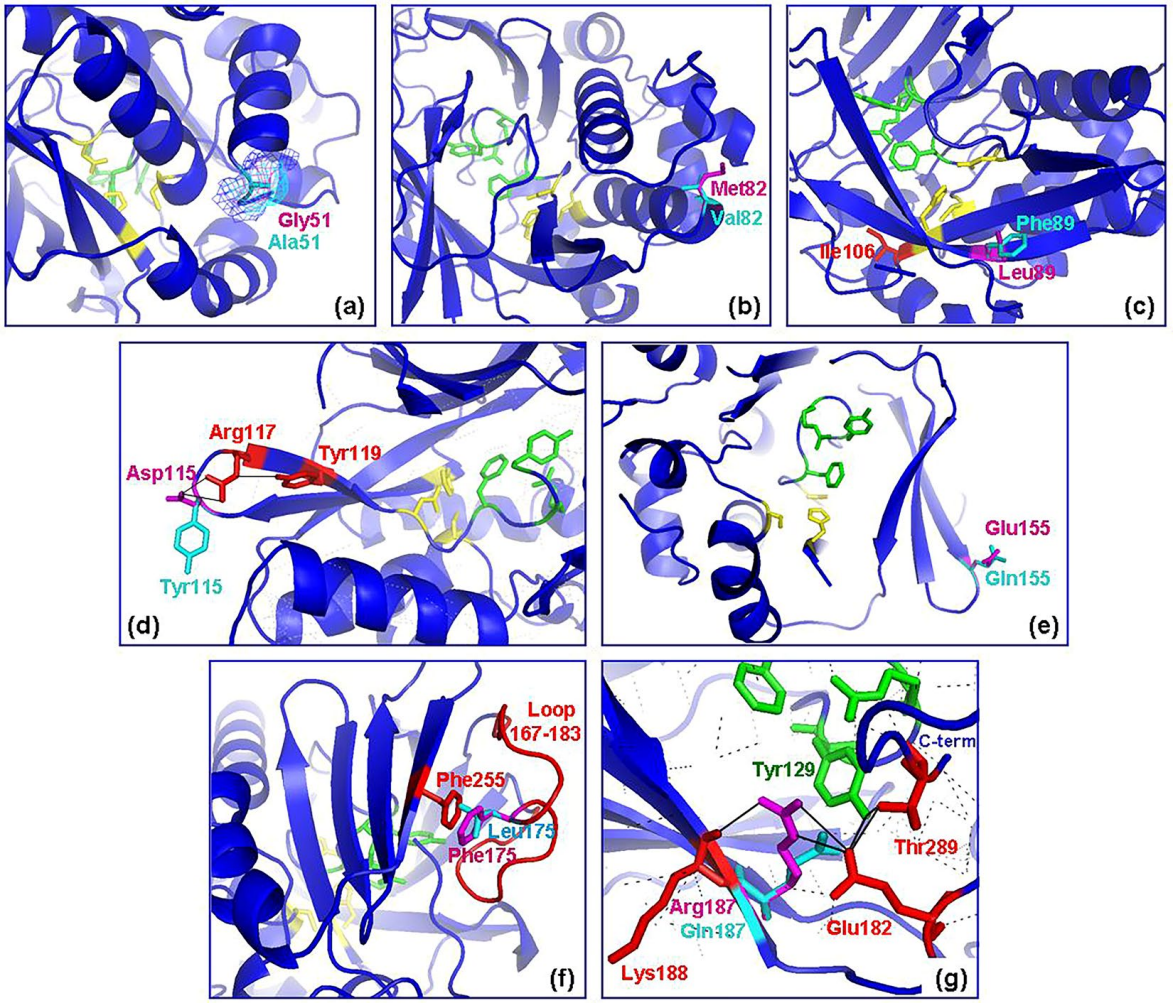
Position of polymorphic residues on (MACMU)NAT1 protein structure. Detailed partial views of the three-dimensional structure of (MACMU)NAT1 protein (blue “cartoon” illustration), showing the position of polymorphisms p.Gly51Ala (**a**), p.Met82Val (**b**), p.Leu89Phe (**c**), p. Asp115Tyr (**d**), p. Glu155Gln (**e**), p.Phe175Leu (**f**) and p.Arg187Gln (**g**). In a-g, magenda and cyan are the polymorphic residues found on the reference and each variant protein, respectively. The residues in yellow are p.Cys68-p.His107-p.Asp122 of the catalytic triad, while residues in green are p.Phe125-p.Arg127-p.Tyr129 of the important catalytic pocket loop 125–129. In a, the “mesh” *vs*. “sticks” representation was preferred for polymorphic residue p.Gly51Ala, to better illustrate the difference in space occupied by the side chain of each amino acid. In c, d, f and g, additional residues or structural elements of relevance are coloured red and marked accordingly. In d and g, the solid black lines indicate important interactions involving the polymorphic residues. The protein was modelled against the known crystallographic structure of (HUMAN)NAT1 (PDB ID: 2PQT)^64^.

Structural alignment and homology modelling of (MACMU)NAT1 protein against the published structure of (HUMAN)NAT1^64^ was used to assess the potential functional effects of rhesus macaque polymorphisms at the molecular level (Figs 6 and S5). These predictions were further compared with the output of PolyPhen-2, a computational algorithm employing comparative sequence alignments and structural analyses to determine the impact of amino acid substitutions on proteins (Table 2).

Residue p.Gly51 is located within the α-helical domain I on the surface of the protein molecule, right before the start of α3-helix and behind p.Cys68 of the catalytic triad. No direct interactions of p.Gly51 are evident and its substitution to Ala is conservative (Figs 6a and S5). Therefore, the postulated effect is benign, which is in line with the prediction of PolyPhen-2 assigning a low impact probability score to this particular polymorphism (Table 2). Despite those computational predictions, the experimental results show p.Gly51Ala to have a moderate effect on (MACMU)NAT1 enzyme activity, presumably by compromising its intrinsic stability. The conformational flexibility of glycine makes the amino acid important for maintaining certain structural arrangements (e.g. tight turns), so its substitution could potentially affect protein stability^95^.

A similar impact can be attributed to polymorphism p.Met82Val which also affects a residue of domain I and is located on the surface of the protein molecule. Amino acid p.Met82 is the last amino acid on α4-helix, which has catalytic p.Cys68 as its first residue (Figs 6b and S5). Substitution from Met to Val is conservative and predicted as benign by PolyPhen-2 (Table 2). The experimental results, however, indicate a moderate effect on enzymatic activity, potentially caused by reduction in intrinsic stability of the protein. Valine has certain restrictions in adopting the α-helical conformation and is hydrophobic, usually found in the interior of protein molecules^95^. Such factors could contribute to the observed instability of variant (MACMU)NAT1_p.Met82Val.

Residue p.Leu89 is located inside the enzymatic core, on β2-strand of β-barrel domain II of the protein molecule^64^. Substitution to Phe is predicted by PolyPhen-2 as probably damaging, with the highest score (Table 2). The same tool identified p.Leu89 as essentially conserved across NAT1 homologues in the UniProt database. The residue is located directly behind β4-strand carrying p.His107 of the catalytic triad and its adjacent p.Ile106 that is important for binding of NAT1-selective aminobenzyl substrates, like p-aminosalicylic acid and PABA^64^. Structural modelling (Fig. 6c) supports that replacement of p.Leu89 by the much bulkier Phe causes a direct steric clash with β4-strand, leading to molecule destabilization and impairement of its catalytic function, consistent with the experimental observations of the previous sections.

Substitution of p.Asp115 by the bulkier, uncharged and more hydrophobic Tyr represents a non-conservative change that was predicted by PolyPhen-2 as possibly damaging to (MACMU)NAT1 (Table 2). Amino acid p.Asp115 is located on the surface of the protein molecule and is part of domain II (Figs 6d and S5). This residue is positioned right at the turn between anti-parallel strands β4 and β5, which determine the positioning of p.His107 and p.Asp122 in the catalytic triad. The conformation of the two β-strands is stabilized via bonding between p.Asp115 and p.Arg117. As described in the previous sections, polymorphism p.Asp115Tyr appeared to compromise the protein molecule in terms of stability and possibly catalytic function. Moreover, the combination of this polymorphism with p.Leu89Phe on the same (MACMU)NAT1 variant was demonstrated to be effectively detrimental. Apart from those two polymorphisms, haplotype (MACMU)NAT1*10 also contained a third SNV causing substitution p.Glu155Gln. However, given the severe impairement of double mutant (MACMU)NAT1_p.Leu89Phe/p.Asp115Tyr, the potential additive effect of the more benign p.Glu155Gln substitution was not examined.

Residue p.Glu155 is the last amino acid on β8-strand of domain II, which is connected to its anti-parallel β9-strand via a sharp turn. The location is away from the catalytic pocket and on the surface of the enzyme molecule (Figs 6e and S5). Its substitution to Gln is conservative and has no apparent consequences. PolyPhen-2 also predicted this change as benign, assigning a very low score (Table 2). Although our experiments detected no substantial impact on enzymatic activity, an apparent decrease in stability of variant (MACMU)NAT1_p.Glu155Gln was nevertheless observed. The underlying cause of this effect is unclear, although p.Glu155 could stabilize the conformation of anti-parallel strands β8-β9, which are part of a β-sheet forming a “wall” on one side of the active site pocket.

Residue p.Phe175 is one of 17 amino acids forming the so-called “eukaryotic loop” in domain II of mammalian NATs. This component is thought to stabilize an important β-sheet of domain III, comprising four anti-parallel β-strands that form a “wall” at the bottom of the active site pocket^64^. This stabilization is conferred via parallel alignment of the loop with the fourth strand (β15) of the β-sheet and, specifically, via “stacking” interaction of the aromatic rings of p.Phe175 on the loop with p.Phe255 on the β15-strand (Fig. 6f). The structural model predicts replacement of p.Phe175 by Leu to be damaging to this interaction and, consequently, to the protein molecule itself, both in terms of stability and potentially enzymatic function. PolyPhen-2 identified p.Phe175 as very conserved across NAT homologues and predicted its substitution as possibly damaging (Table 2). The *in silico* results are consistent with the experimental observations of the previous sections.

The final polymorphism in this study, p.Arg187Gln, has been structurally modelled before, as it is also found in (HUMAN)NAT1^64,88,96,97^. As shown (Figs 6g and S5), residue p.Arg187 is located on β11-strand of domain II and forms hydrogen bond interactions with p.Lys188 and p.Glu182. The latter amino acid is located on the aforementioned “eukaryotic loop” of the enzyme and interacts via hydrogen bonding with two important residues, p.Tyr129 and p.Thr289. Residue p.Tyr129 is key for NAT1 acceptor substrate selectivity^80^ and also interacts with p.Thr289, which is the residue located just before the carboxyl terminus of the 290 amino acid polypeptide chain. This network of interactions maintains the shape of the active site pocket and of the “eukaryotic loop”, while also stabilizing the carboxyl terminus in a position deep within the catalytic core of the enzyme that enables contact with the donor substrate^64^. Although the rather conservative substitution of p.Arg187 to Gln could potentially maintain some of the required hydrogen bond interactions, instability in the polypeptide chain folding would be unavoidable, in line with the moderate loss of protein observed during recombinant expression/purification. Moreover, the expected change in the conformation of the active site pocket would explain the substantial decrease in enzymatic activity observed for variant p.Arg187Gln both in the rhesus macaque and human. Contrary to the solid experimental evidence, PolyPhen-2 predicted this polymorphism as benign, although the score was close to 0.5 (Table 2). Despite their value in guiding the assessment of SNV effects on protein function, computational predictions must always be interpreted with caution, as they may not be fully aligned with experimental evidence.

## Discussion

The evolution of xenobiotic metabolizing enzymes is shaped by the ever-changing chemical environments organisms need to adapt to in order to survive. Genetic polymorphisms can be favourable, in that respect, as they confer a range of metabolic activities towards a plethora of potentially harmful xenobiotic substances. Moreover, population frequencies of high- *vs*. low-activity enzyme variants may shift to one or the other direction, depending on the prevailing xenobiotic challenges in a specific environment. In humans, this process is driven not only by natural selective pressures, but also by the lifestyle choices of our species. The NAT enzymes metabolize toxic aromatic amines found in cooked food and other byproducts of everyday human activity since prehistoric times. Variability in *NAT* genes has thus been studied in the context of understanding how subsistence patterns may have affected the genetic makeup of human populations^58,59,98–103^. Such knowledge is useful for elucidating how even apparently modest differentiations in gene-environment interactions may influence disease susceptibility or drug response.

Comparison of humans with other primates can further discriminate between variability that is ancestral or specific to our own species. Although previous phylogenetic analyses have demonstrated a dynamic pattern consistent with adaptive evolution of *NAT* genes in primates^62,63^, those studies have been limited by their entirely *in silico* methodology and the fact that they have examined *NAT* variability only between species. A new study, investigating population variability at *NAT1*, *NAT2* and *NATP* loci across different hominid species, demonstrated opposite diversity patterns for the chimpanzee (*Pan troglodytes*) and bonobo (*P*. *paniscus*) compared with human. Genetic diversity was higher for *NAT1* in the two *Pan* species and lower in human, with the opposite pattern observed for the *NAT2* gene^60^. Here, we investigated *NAT1* variability in a population sample of the Old World monkey rhesus macaque, also characterizing the possible functional consequences of detected variants. Our results are in accordance with the aforementioned study^60^, indicating high haplotypic diversity (Hd = 0.817) of (MACMU)*NAT1* coding region for the specific rhesus macaque population studied, and with 16% of the haplotypes carrying at least one non-synonymous SNV with some functional impact. It appears that, in the rhesus macaque, polymorphic *NAT1* alleles may modulate acetylation of arylamine xenobiotics more effectively compared with human, where population frequencies of non-synonymous SNVs in *NAT1* coding region are extremely low (typically <1‰, with few exceptions)^104^. The low haplotypic diversity (Hd = 0.073) reported for (HUMAN)*NAT1* coding region is consistent with the action of purifying selection predicted for the gene^58^. As mentioned above, (HUMAN)*NAT1* is considered to have a more fundamental role in cells, unlike its paralogous (HUMAN)*NAT2* which is higly polymorphic and exhibits the functional characteristics of a typical xenobiotic metabolizing enzyme.

Although (MACMU)*NAT1* is phylogenetically orthologous to (HUMAN)*NAT1*^62^, its higher degree of variability could be the outcome of environmental pressures differentiating the evolutionary lineages of the two species. However, despite this high level of diversity, all heterozygous (MACMU)*NAT1* genotypes were found to contain at least one allele with no predicted phenotypic consequences. Specifically, of the 25 genotypes determined, seventeen contained only functional alleles (i.e. combinations of *1, *3, *5, *8, *9 and *12 alleles encoding for the reference amino acid sequence), while the remaining eight genotypes combined one functional with one compromised (*2, *4, *6, *7, *10, *11) allele. Genotypes consisting of two compromised alleles were not found (Supplementary Table S1). It is possible that rhesus macaque *NAT1* variability may be maintained, as far as the corresponding enzymatic function is not entirely abolished in the individual. This could preserve the same endogenous function attributed to (HUMAN)NAT1, although the diversity pattern appears to differ in the evolutionary lineages of the two species. Although such observations are useful, in view of current hypotheses relating (HUMAN)*NAT1* with functions beyond xenobiotic metabolism, other factors (potentially related to the small size or the particular makeup of the rhesus macaque population from which our sample was drawn) could also account for the high variability observed.

As described above, (HUMAN)*NAT1* is also differentiated from other primate orthologues at the 3′-UTR of the gene. The reference allelic sequence downstream of the coding region bears a microsatellite repeat element that encompasses an active polyA signal of the gene. Variability in this element is common (~40% global frequency) in human populations, and is encountered both in the form of SNV, e.g. rs1057126:T > A in (HUMAN)NAT1*10, and in the form of simple sequence length polymorphism, as in (HUMAN)NAT1*11 and other alleles. Although those variations appear to confer some functional effects, their association with disease susceptibility has been controversial^2,72^. It would be interesting to examine if the functional significance attributed to the 3′-UTR of (HUMAN)*NAT1* gene is corroborated by studies with *NAT1* orthologues of other primates, given the substantial sequence and structural variability we report here.

Despite those differences, certain *NAT1* variants are found in both the rhesus macaque and human. As discussed, *NAT1* polymorphism c.560G > A (p.Arg187Gln) was the most prevalent non-synonymous SNV in our rhesus macaque sample and is relatively common in human populations too. The functional impact of this polymorphism was similar for the two species, compromising the activity of NAT1 isoenzyme. Other rhesus macaque SNVs of this study have also been reported for (HUMAN)*NAT1* gene by large scale genome-wide population studies, like the 1000 Genomes Project, including synonymous SNVs at positions c.15 (rs747456038:A > G) and c.321 (rs755240678:C > T), as well as the non-synonymous SNV at position c.523 (rs762246777:T > C; p.Phe175Leu). Moreover, similarly to the characterized (MACMU)*NAT1* variants, impaired intrinsic stability appears to be the main defect of low-activity (HUMAN)*NAT1* variants, such as NAT1*17 (c.190C > T, p.Arg64Trp; rs56379106:C > T) and *22 (c.752A > T, p.Asp251Val; rs56172717:A > T)^23,70,71,88,93^.

Although pinpointing genetic aberrations that are strongly associated with disease is relatively straightforward, it is more difficult to unravel subtle genomic differences that may influence adaptability to exogenous challenges and shape the landscape of factors contributing to complex disorders. Xenobiotic metabolizing enzymes play diverse roles in cells that may not be limited merely to protection of organisms from exogenous substances. Therefore, any link to disease, particularly carcinogenesis, may be either via modulation of the mutagenic potential of xenobiotics, or via a less obvious interference with cell physiology, as has long been speculated for human *NAT1* and is supported by recent studies mentioned above. Comparative genetic analyses between human and other primates add the evolutionary dimension to such investigations, particularly when combined with experimental insights into functions that may have been either conserved or diversified in the phylogenetic lineages separating our species from its closest relatives. To that end, it would be useful to conduct more comprehensive interdisciplinary investigations into the molecular, structural and enzymological aspects of variants reported for primate NAT homologues.

## Methods

### Amplification and sequencing of (MACMU)*NAT1* gene

For this *in vitro* study, the 870 bp intronless coding region of (MACMU)*NAT1* gene was amplified from genomic DNA samples of a rhesus macaque population described previously^105,106^. PCR was performed with flanking primers 1 A (5′-AGCCATAATTAGCCTACTC-3′) and 1AR (5′-GTACAGAAGATACATGATAGG-3′), and the reactions contained 1 u GoTaq DNA polymerase (Promega) in 1x reaction buffer with 1.5 mM MgCl_2_, 0.1 mM each dNTP, 0.1 μM each primer and 25 ng genomic DNA as template. Thermal cycling conditions were as follows: 95 °C (10 min), followed by 38 cycles of 94 °C (1 min), 53 °C (50 s) and 72 °C (2 min), with final extension at 72 °C (10 min). Specific PCR amplification was verified by agarose gel electrophoresis and the products (1350 bp) were purified and sequenced from both directions (Millegen, Labège, France).

### Generation of polymorphic (MACMU)*NAT1* constructs

To date, the *NAT1* homologues of three macaque species (*M*. *mulatta*, *M*. *sylvanus* and *M*. *fascicularis*) have been cloned and characterized^61,107^, and those encode identical polypeptide chains. The (MACMU)*NAT1* reference sequence (Nucleotide ID: KU640969.1) of the rhesus macaque was already available^61^ in pET28b(+) expression vector (Novagen) for the purposes of this study. Incorporation of each non-synonymous SNV found in the various (MACMU)*NAT1* haplotypes was performed by site-directed mutagenesis using the QuikChange II kit (Agilent), as previously described^74^. The mutagenised constructs were verified by sequencing of the insert (GATC Biotech, Konstanz, Germany).

### Recombinant protein expression and purification

Expression of polyhistidine-tagged (MACMU)NAT1 protein variants was carried out in 200 ml cultures of *E*. *coli* BL21(DE3)pLysS cells as previously described^61,73^. The soluble fraction of bacterial lysates was recovered and immediately subjected to purification by affinity chromatography through Nickel-charged columns (Qiagen). Recombinant proteins were eluted with increasing concentration of imidazol and analysed by quantification (280 nm) on a Nanodrop 2000 Spectrophotometer (Thermo Scientific). Protein separation was carried out by sodium dodecyl sulphate polyacrylamide gel electrophoresis (SDS-PAGE) and gels were photographed with BioRad ChemiDoc XRS + Imaging System. All handling of proteins took place on ice or at 4 °C, and subsequent experiments were carried out within one day from protein production (while maintaining on ice in a cold chamber), to avoid freeze-thawing.

### Immunoblot analysis

Immunoblot detection was performed according to standard practices, using the BioRad Mini-Protean Tetra Cell System and Criterion Blotter. Gels were blotted onto Immuno-Blot PVDF membrane for antibody binding and subsequent chemiluminescence detection with Immun-Star Western kit (both products from BioRad). The antibodies used were: (a) commercial horseradish peroxidase (HRP) conjugated monoclonal antibody A7058 (Sigma-Aldrich) against the polyhistidine tag of recombinant proteins, diluted 1:12,000; (b) commercial non-selective NAT rabbit polyclonal antibody ARP44197 (kindly provided by Aviva Systems Biology, San Diego, CA, USA), diluted 1:2000; c) NAT1-selective rabbit polyclonal antiserum #183, raised against the bovine serum albumin (BSA) conjugated C-terminal peptide of human NAT1 (kindly provided by Professor Edith Sim, University of Oxford, UK), diluted 1:4000. The binding of antibodies ARP44197 and #183 was detected with HRP-conjugated monoclonal anti-rabbit IgG secondary antibody A1949 (Sigma-Aldrich), diluted 1:10,000. Quantification of affinity chromatography purified proteins (100 mM imidazol fractions) was carried out by spectrophotometry at 280 nm (Nanodrop). Total protein quantification in cell lysates was carried out using the Bradford reagent (Sigma-Aldrich), as instructed by the manufacturer. The ChemiDoc XRS + Imaging System was used to detect and quantify the chemiluminescent signal on immunoblot membranes.

### Enzyme activity assays

The enzyme activity assays^108^ were performed with 1 μg of affinity chromatography purified NAT1 protein (100 mM imidazol fraction), according to well-standardized procedures in our laboratory^61,73,74^. The acyl-group acceptor substrates used (0.5 mM) were the NAT1-selective PABA (PubChem CID: 978) and 5AS (PubChem CID: 4075), the NAT2-selective SMZ (PubChem CID: 5327), all three pharmaceutical arylamines, and the less selective PANS (PubChem CID: 7732), a toxic arylamine. Acetyl-CoA (0.4 mM) was used as acyl-group donor substrate and the enzymatic release of CoA was monitored over time with Ellman’s reagent. Reactions were performed in duplicate.

### Thermal stability assays

For comparing thermal stability of recombinant (MACMU)NAT1 variants of the rhesus macaque, protein expression/purification was carried out and the enzyme activity was recorded using PABA as substrate. The procedures were as described in the previous sections. Each recombinant protein preparation was then incubated for 10 min on a PCR DNA Engine Tetrad Gradient Cycler (MJ Research) across a temperature range spanning the denaturation midpoint temperature (Tm = 36.5 ± 1.2 °C) previously determined for (MACMU)NAT1 recombinant protein by differential scanning fluorimetry^61^. Following thermal stress, the activity of each recombinant variant was measured with PABA during a 5 min enzymatic assay, as described above.

### Computational analyses

BioEdit version 7.1.9^109^ was used to inspect sequences and perform multiple alignments on ClustalW. *NAT1* haplotypes were inferred from the unphased genotypes of sequenced individuals with PHASE v2.1.1 software^110^, using the default parameter values in the Markov chain Monte Carlo simulations. The algorithm was applied ten times with different random seed numbers to check for consistency of the results across the independent runs. We chose the results from the run displaying the best average goodness-of-fit of the estimated haplotypes to the underlying coalescent model. Primate sequences for NAT1 were retrieved from the Genome Database (https://www.ncbi.nlm.nih.gov/genome/), using BLASTn, and the search for repetitive elements downstream of *NAT1* gene was carried out with RepeatMasker open version 4.0.8 (http://www.repeatmasker.org). The presentation of SNVs in the manuscript is according to the guidelines of the Human Genome Variation Society (http://www.hgvs.org/mutnomen/) and each haplotype was named following the recommendations of the *NAT* Gene Nomenclature Committee^111^ (http://nat.mbg.duth.gr/). The reference allele^61^ (Nucleotide ID: KU640969.1) was assigned symbol (MACMU)NAT1*1 and the remaining haplotypes were named in the order of their annotation during the study. A GenBank accession number (Nucleotide ID) was assigned per haplotype.

Structural alignment of protein sequences was carried out with T-COFFEE Expresso (http://tcoffee.crg.cat/apps/tcoffee/do:expresso) and graphically visualised with ESPript3.0 (http://espript.ibcp.fr). Modelling of (MACMU)NAT1 was performed against the known structure of human NAT1 protein (PDB ID: 2PQT)^64^, using the Swiss-Model online tool (http://swissmodel.expasy.org/). Graphical visualisation of models was performed on PyMOL (Schrödinger, LLC). The effect of each polymorphism on (MACMU)NAT1 protein was also predicted using PolyPhen-2 (HumDiv model, http://genetics.bwh.harvard.edu/pph2/), an online bioinformatics tool assigning impact probability scores in the scale from 0.00 (benign) to 1.00 (probably damaging). The potential consequences of amino acid substitutions were further assessed according to Russell and colleagues^95^ (http://www.russelllab.org/aas/).

## Supplementary information

Supplementary Information AND Expanded Data
