# Supplementary material for: Population variability of rhesus macaque (Macaca mulatta) NAT1 gene for arylamine N-acetyltransferase 1: Functional effects and comparison with human
Source: Sci Rep. 2019 Jul 29;9:10937. doi: 10.1038/s41598-019-47485-x (PMC6662693; doi:10.1038/s41598-019-47485-x)
Supplement: Supplementary file 1 — Supplementary Information AND Expanded Data [file 41598_2019_47485_MOESM1_ESM.pdf]

Supplementary Information

&

Expanded Data Supplementary Figure

To manuscript entitled:

Population variability of rhesus macaque (*Macaca mulatta*) *NAT1* gene for arylamine *N*-acetyltransferase 1: Functional effects and comparison with human

By authors:

Sotiria Boukouvala, Zoi Chasapopoulou, Despina Giannouri, Evanthia Kontomina, Nikolaos Marinakis, Sophia V. Rizou, Ioanna Stefani, Theodora Tsirka, Charlotte Veyssière, Sofia Zaliou, Audrey Sabbagh, Brigitte Crouau-Roy, Giannoulis Fakis

Supplementary Table S1: Genotyping of 25 rhesus macaque individuals for nucleotide variation in the coding region of (MACMU)*NAT1* gene[a,b].

Genotyped individuals	Nucleotide viaration											
	c.15	c.152	c.177	c.244	c.267	c.321	c.343	c.463	c.493	c.523	c.540	c.560	
Reference	A	G	C	A	G	C	G	G	A	T	C	G	Allelic combination
Ma1	A/A	G/G	C/C	**A/G**	G/G	**C/T**	G/G	G/G	A/A	T/T	**C/T**	G/G	*NAT1*1/*2*
Ma3	A/A	**G/C**	C/C	A/A	G/G	**T/T**	G/G	G/G	A/A	T/T	C/C	G/G	*NAT1*3/*4*
Ma5	A/A	G/G	C/C	A/A	G/G	**T/T**	G/G	G/G	A/A	T/T	**T/T**	G/G	*NAT1*5/*5*
R26	A/A	G/G	C/C	A/A	G/G	**C/T**	G/G	G/G	A/A	T/T	**C/T**	G/G	*NAT1*1/*5*
R27	A/A	G/G	C/C	A/A	G/G	**C/T**	G/G	G/G	A/A	T/T	**C/T**	G/G	*NAT1*1/*5*
R28	A/A	G/G	C/C	A/A	G/G	**C/T**	G/G	G/G	A/A	**T/C**	**C/T**	G/G	*NAT1*1/*6*
R29	A/A	G/G	C/C	A/A	G/G	**C/T**	G/G	G/G	A/A	T/T	C/C	G/G	*NAT1*1/*3*
R30	A/A	G/G	C/C	A/A	G/G	**C/T**	G/G	G/G	A/A	T/T	C/C	**G/A**	*NAT1*1/*7*
R31	A/A	G/G	C/C	A/A	G/G	**C/T**	G/G	G/G	A/A	T/T	**C/T**	G/G	*NAT1*1/*5*
R32	**G/G**	G/G	C/C	A/A	G/G	C/C	G/G	G/G	A/A	T/T	C/C	G/G	*NAT1*8/*8*
R33	A/A	G/G	C/C	A/A	G/G	**T/T**	G/G	G/G	**C/C**	T/T	C/C	G/G	*NAT1*9/*9*
R34	A/A	G/G	C/C	A/A	G/G	**C/T**	G/G	G/G	A/A	T/T	C/C	G/G	*NAT1*1/*3*
R35	A/A	G/G	C/C	A/A	G/G	C/C	G/G	G/G	A/A	T/T	C/C	G/G	*NAT1*1/*1*
R36	A/A	G/G	C/C	A/A	G/G	**C/T**	G/G	G/G	**A/C**	T/T	C/C	G/G	*NAT1*1/*9*
R37	A/A	G/G	C/C	A/A	**G/C**	**C/T**	**G/T**	**G/C**	A/A	T/T	C/C	G/G	*NAT1*1/*10*
R38	A/A	G/G	C/C	A/A	G/G	**C/T**	G/G	G/G	A/A	T/T	C/C	**G/A**	*NAT1*1/*7*
R39	A/A	G/G	**C/T**	A/A	G/G	**T/T**	G/G	G/G	A/A	T/T	**C/T**	G/G	*NAT1*5/*12*
R40	A/A	G/G	C/C	A/A	G/G	**T/T**	**G/T**	G/G	A/A	T/T	**C/T**	G/G	*NAT1*5/*11*
R41	A/A	G/G	C/C	A/A	G/G	**C/T**	G/G	G/G	A/A	T/T	C/C	G/G	*NAT1*1/*3*
R42	A/A	G/G	C/C	A/A	G/G	**C/T**	G/G	G/G	A/A	T/T	C/C	**G/A**	*NAT1*1/*7*
R44	A/A	G/G	C/C	A/A	G/G	**T/T**	G/G	G/G	A/A	T/T	**C/T**	G/G	*NAT1*3/*5*
R46	A/A	G/G	C/C	A/A	G/G	**T/T**	G/G	G/G	A/A	T/T	**C/T**	G/G	*NAT1*3/*5*
R47	A/A	G/G	C/C	A/A	G/G	**C/T**	G/G	G/G	A/A	T/T	C/C	G/G	*NAT1*1/*3*
R48	A/A	G/G	C/C	A/A	G/G	**T/T**	G/G	G/G	A/A	T/T	**C/T**	G/G	*NAT1*3/*5*
R49	A/A	G/G	C/C	A/A	G/G	**C/T**	G/G	G/G	A/A	T/T	C/C	G/G	*NAT1*1/*3*

[a]Detected variant sites are shown, numbered relative to the intronless coding region of (MACMU)*NAT1* gene (position c.1 corresponds to the adenosine of the ATG translation initiation codon). The reference allele is (MACMU)*NAT1*1* with Nucleotide ID: KU640969.1 [61]. Variant alleles **2*-**12* were named according to their order of annotation. Genotypic variation is indicated with bold font and the inferred allelic combinations are reported.

[b]The study was conducted entirely *in vitro*, using a legacy collection of genetic material originating from a Chinese rhesus macaque population described in earlier studies [105,106]. The Hardy-Weinberg equilibrium test for this population showed no apparent deviation.

Supplementary Table S2: Description of (MACMU)*NAT1* polymorphic constructs expressed in this study.

Construct name	Non-synonymous SNV(s)	Amino acid change(s)	Mutagenesis primers[a]
(MACMU)NAT1_1	Reference sequence	None	Construct available
(MACMU)NAT1_p.Gly51Ala	c.152G>C	p.Gly51Ala	1F: gaagccatggacttag**c**cttagaggccattttg<br>1R: caaaaatggcctctaag**g**ctaagtccatggcttc
(MACMU)NAT1_p.Met82Val	c.244A>G	p.Met82Val	2F: ggctctgaccact**g**tgggttttgagaccac<br>2R: gtggtctcaaaaccca**c**agtggtcagagcc
(MACMU)NAT1_p.Leu89Phe	c.267G>C	p.Leu89Phe	3F: gagaccacgatgtt**c**ggagggtatgtttacaac<br>3R: gttgtaaacataccctcc**g**aacatcgtggtctc
(MACMU)NAT1_p.Asp115Tyr	c.343G>T	p.Asp115Tyr	4F: cctgcaggtgaccatt**t**acggcaggaactac<br>4R: gtagttcctgccgt**a**aatggtcacctgcagg
(MACMU)NAT1_p.Leu89Phe/p.Asp115Tyr	c.267G>C and c.343G>T	p.Leu89Phe and p.Asp115Tyr	SNV c.343G>T (p.Asp115Tyr) was introduced to construct (MACMU)NAT1_p.Leu89Phe using mutagenesis primers 4F+4R
(MACMU)NAT1_p.Glu155Gln	c.463G>C	p.Glu155Gln	5F: cttccgcttgacggaa**c**agaatggattctgg<br>5R: ccagaatccattct**g**ttccgtcaagcggaag
(MACMU)NAT1_p.Phe175Leu	c.523T>C	p.Phe175Leu	6F: ccagtacattccaaataaagaa**c**ttcttaattctgatcttctag<br>6R: ctagaagatcagaattaagaa**g**ttctttatttggaatgtactgg
(MACMU)NAT1_p.Arg187Gln	c.560G>A	p.Arg187Gln	7F: ctagaagacagcaaatacc**a**aaaaatctactcctttac<br>7R: gtaaaggagtagattttt**t**ggtatttgctgtcttctag

[a]F is forward and R is reverse primer, combined together during site-directed mutagenesis experiments to introduce each SNV to the cloned [61] (MACMU)*NAT1* reference sequence. The mutagenised site is shown in bold. All primers were from VBC Biotech.

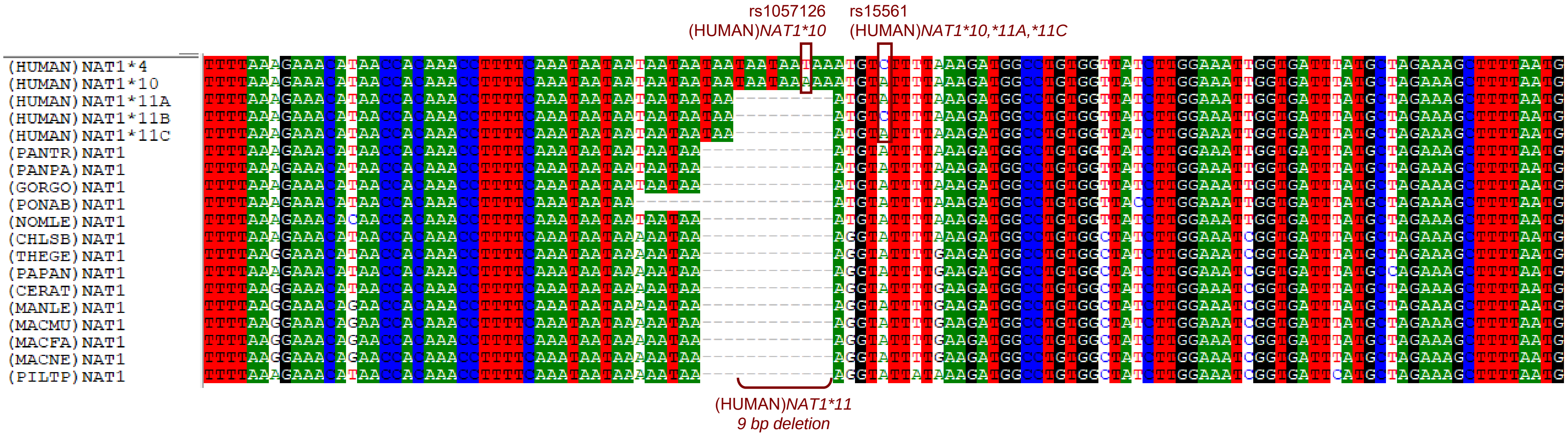

Supplementary Figure S1: Alignment of primate *NAT1* genomic sequences around the variable region of (HUMAN)*NAT1*10* and (HUMAN)*NAT1*11* alleles, commonly found in human populations.

Shown (first line of the alignment) is the genomic sequence at 161-283 bp after the stop codon of (HUMAN)*NAT1*4* reference allele. The corresponding sequences of (HUMAN)*NAT1*10* and **11A,B,C* polymorphic alleles are aligned to the reference human allele in lines 2-5. SNVs 1088T>A (c.*215T>A, rs1057126) and 1095C>A (c.*222C>A, rs15561) defining the (HUMAN)*NAT1*10* allele, as well as the characteristic 9 bp deletion defining (HUMAN)*NAT1*11* alleles, are marked. The remaining lines of the alignment show the corresponding *NAT1* sequences of the apes (Hominoidea) *Pan troglodytes* (PANTR), *Pan paniscus* (PANPA), *Gorilla gorilla* (GORGO), *Pongo abelii* (PONAB) and *Nomascus leucogenys* (NOMLE), as well as of the Old World monkeys (Cercopithecidae) *Chlorocebus sabaeus* (CHLSB), *Theropithecus gelada* (THEGE), *Papio anubis* (PAPAN), *Cercopithecus atys* (CERAT), *Mandrillus leucophaeus* (MANLE), *Macaca mulatta* (MACMU), *Macaca fascicularis* (MACFA), *Macaca nemestrina* (MACNE) and *Piliocolobus tephrosceles* (PILTP). The sequences were retrieved from the Genome database (NCBI), via BLASTn search against the sequence of (HUMAN)*NAT1*4* reference allele, and aligned with BioEdit v.7.1.9.

Note that the $(TAA)_n$ microsatellite repeat region is shorter in the non-human primates, compared with (HUMAN)*NAT1*4* and other reported human alleles (http://nat.mbg.duth.gr/).

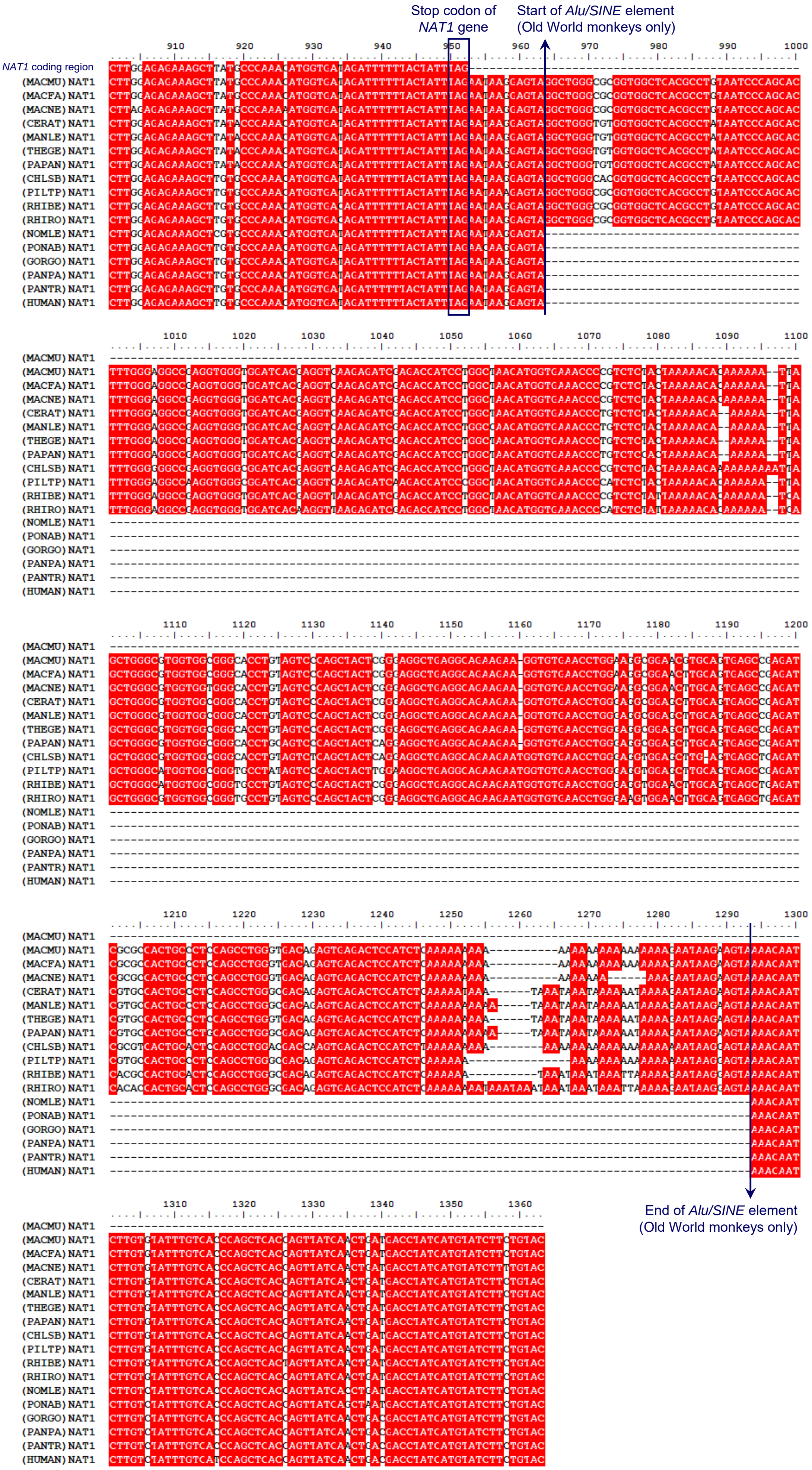

Supplementary Figure S2: Alignment of primate genomic sequences immediately downstream of the *NAT1* gene coding region.

The first line of the alignment shows the end part of (MACMU)*NAT1* gene coding region. The remaining alignment shows the same part of *NAT1* coding region (the stop codons are boxed), plus an extended portion of the downstream genomic sequence, for 17 primate species. The sequences are aligned in the following order: Old World monkeys (Cercopithecidae) *Macaca mulatta* (MACMU), *Macaca fascicularis* (MACFA), *Macaca nemestrina* (MACNE) *Cercopithecus atys* (CERAT), *Mandrillus leucophaeus* (MANLE), *Theropithecus gelada* (THEGE), *Papio anubis* (PAPAN), *Chlorocebus sabaeus* (CHLSB), *Piliocolobus tephrosceles* (PILTP), *Rhinopithecus bieti* (RHIBE) and *Rhinopithecus roxellana* (RHIRO), as well as the apes (Hominoidea) *Nomascus leucogenys* (NOMLE), *Pongo abelii* (PONAB), *Gorilla gorilla* (GORGO), *Pan paniscus* (PANPA), *Pan troglodytes* (PANTR) and *Homo sapiens* (HUMAN). The sequences were retrieved from the Genome database (NCBI), via BLASTn search against the sequence of (MACMU)*NAT1* coding and downstream genomic region, and aligned with BioEdit v.7.1.9.

Note that Old World monkeys carry an insertion of 310-330 bp, which is predicted by RepeatMasker (http://www.repeatmasker.org) to be a typical *Alu/SINE* repeat element. This element is absent in the downstream region of *NAT1* orthologues in apes, including human.

From left to right:

Cell pellets after harvest:

(MACMU)NAT1_p.Glu155Gln
(MACMU)NAT1_p.Met82Val
(MACMU)NAT1_1

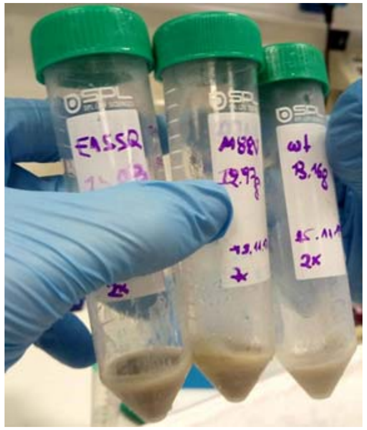

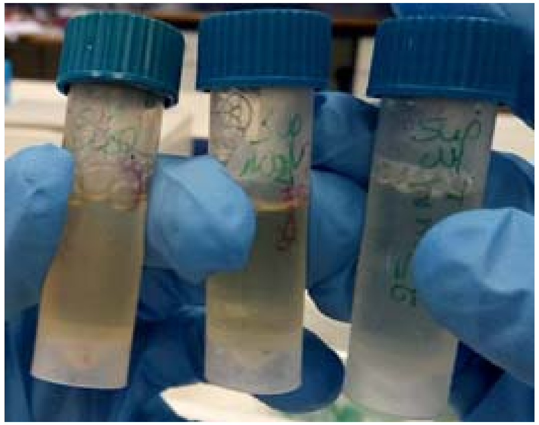

From left to right:

Soluble cell extracts:

(MACMU)NAT1_p.Glu155Gln
(MACMU)NAT1_p.Met82Val
(MACMU)NAT1_1

Cell pellets after harvest:

(MACMU)NAT1_p.Phe175Leu
(MACMU)NAT1_p.Arg187Gln
(MACMU)NAT1_1

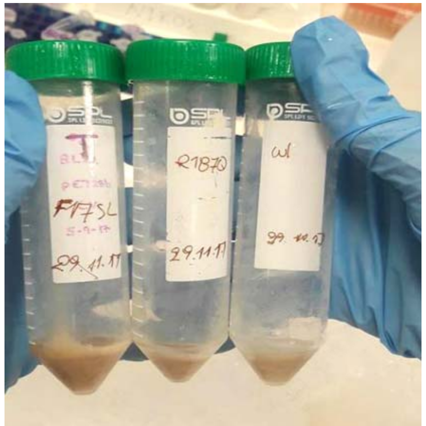

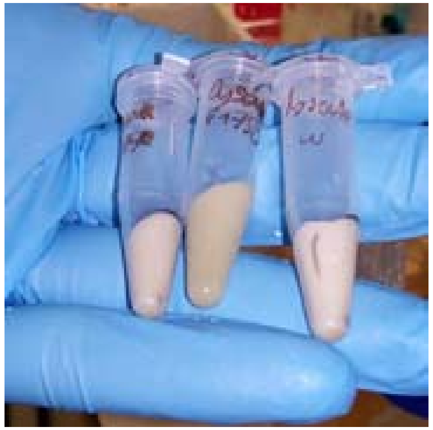

Insoluble cell extracts:

(MACMU)NAT1_p.Arg187Gln
(MACMU)NAT1_p.Phe175Leu
(MACMU)NAT1_1

Cell pellets after harvest:

(MACMU)NAT1_p.Glu115Tyr
(MACMU)NAT1_p.Phe175Leu
(MACMU)NAT1_1

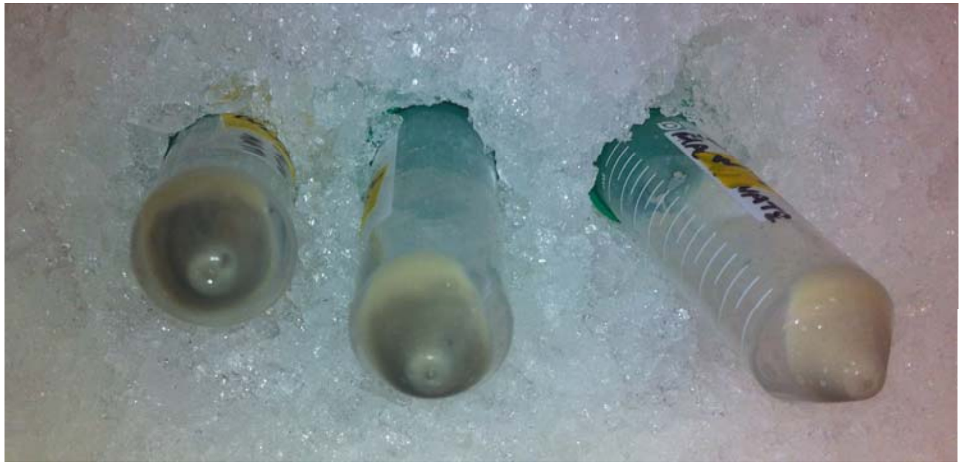

Supplementary Figure S3: Photographs showing differences between (MACMU)NAT1_1 and its variants, during successive steps of recombinant protein preparation.

Note that, with the exception of (MACMU)NAT1_p.Arg187Gln, all preparations of variants were characteristically darker in colour relative to (MACMU)NAT1_1.

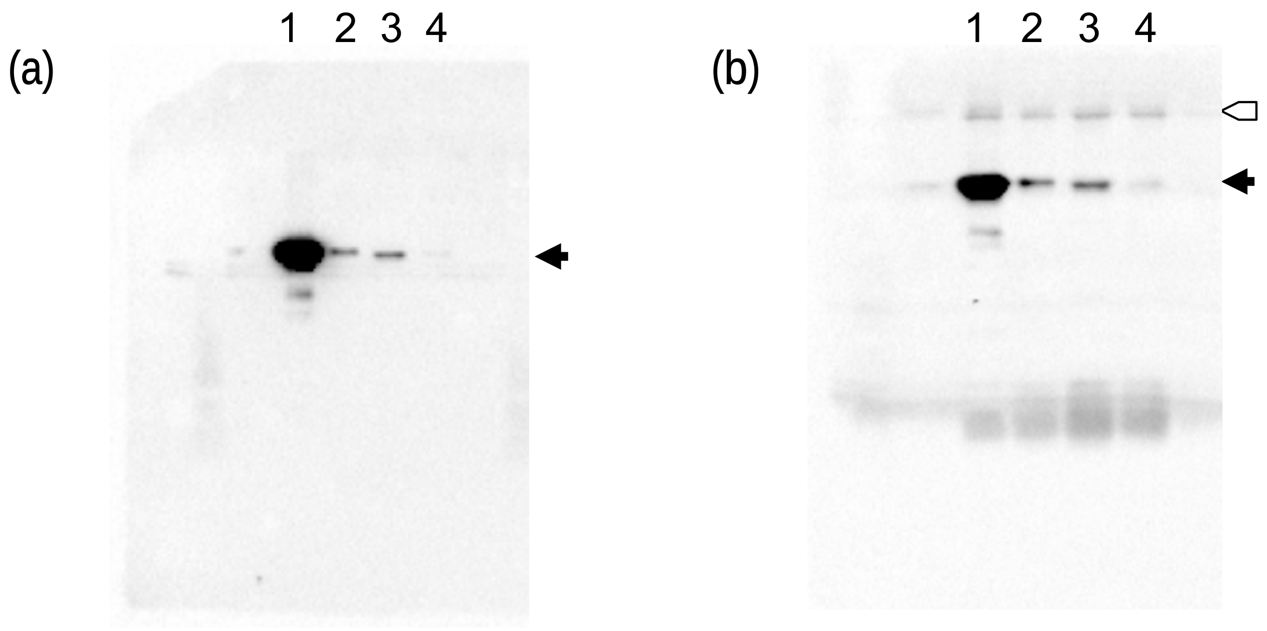

Supplementary Figure S4: Immunodetection of (MACMU)NAT1_p.Leu89Phe/p.Asp115Tyr double variant.

Reference protein (MACMU)NAT1_1 (lane 1) was expressed alongside variants (MACMU)NAT1_p.Leu89Phe (lane 2), (MACMU)NAT1_p.Asp115Tyr (lane 3), and the double mutant (MACMU)NAT1_p.Leu89Phe/p.Asp115Tyr (lane 4). Immunodetection was carried out with antibodies A7058 (a) and #183 (b), using total cell lysates after recombinant expression (0.2 mg total protein per lane). In both images, black arrows indicate the bands of recombinant NAT1 proteins. In b, the white arrow shows the BSA protein (0.2 µg per lane) added to each preparation as control of homogenous loading of samples onto the gel (polyclonal antiserum #183 was raised against a BSA-conjugated NAT1 peptide and thus detects both proteins). Multiple exposures of full-length blots are presented in Expanded Data Supplementary Fig. 1 at the end of the present Supplementary Information file.

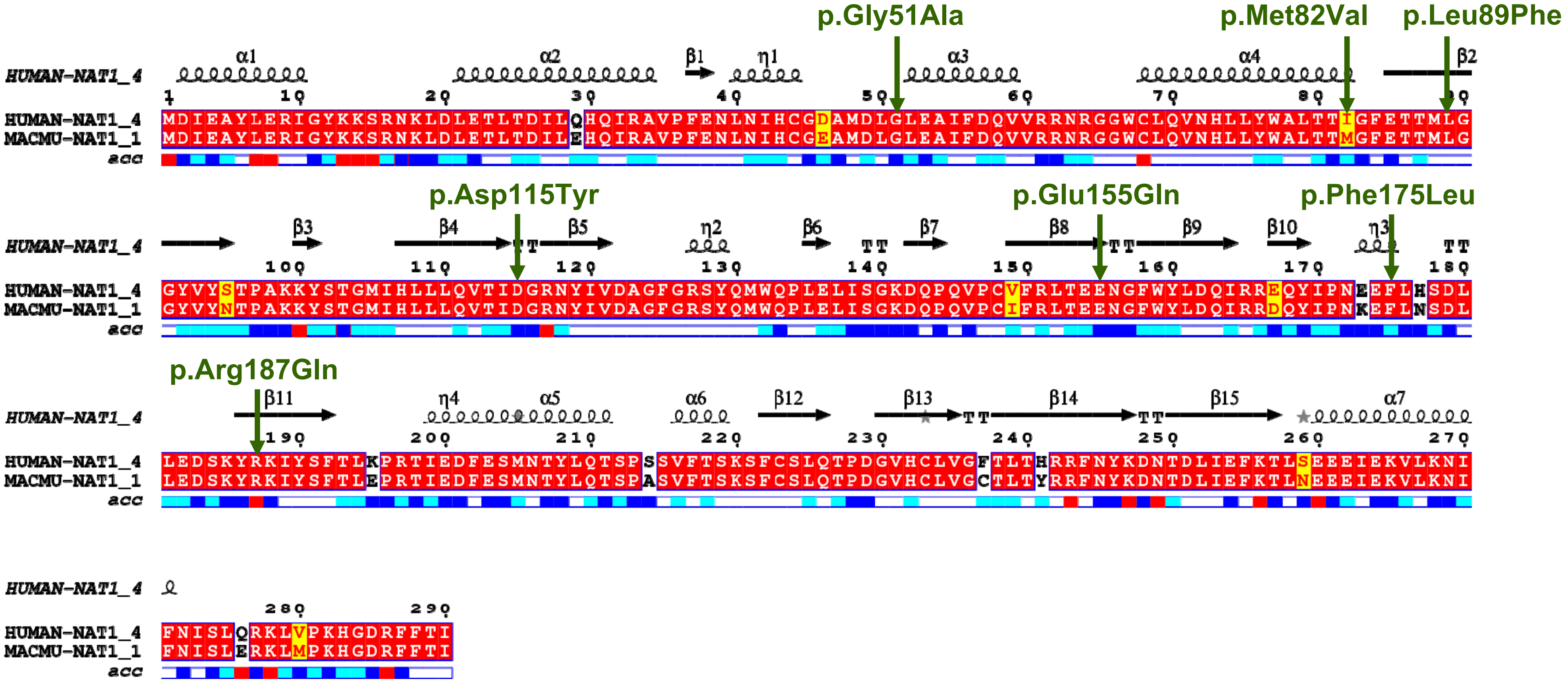

Supplementary Figure S5: Structural alignment of human and rhesus macaque NAT1 protein sequences.

The reference amino acid sequences (HUMAN)NAT1_4 of *Homo sapiens* and (MACMU)NAT1_1 of *Macaca mulatta* were aligned to the secondary structural elements of the former protein (PDB ID: 2PQT). Vertical arrows (green) indicate the positions of residues identified as polymorphic in (MACMU)NAT1. Below the alignment, residues are predicted as accessible (blue), partly accessible (cyan) or buried (white), while red is undetermined. The structural alignment was performed with T-COFFEE Expresso (http://tcoffee.crg.cat/apps/tcoffee/do:expresso) and graphically visualised with ESPript3.0 (http://espript.ibcp.fr).

Expanded Data Supplementary Figure 1

Figure legend: Full-length images of illustrations shown in the main manuscript and supplementary information file, presented in the following order:

Figure 2a (top) of main manuscript
Figure 2a (bottom) of main manuscript
Figure 2b of main manuscript
Figure 3a of main manuscript
Figure 3b of main manuscript
Figure 3c of main manuscript
Figure 3d of main manuscript
Supplementary Figure S4a of Supplementary Information file
Supplementary Figure S4b of Supplementary Information file

Multiple exposure images are provided for all blots.

Full-length image of gel shown in Fig. 2a (top)

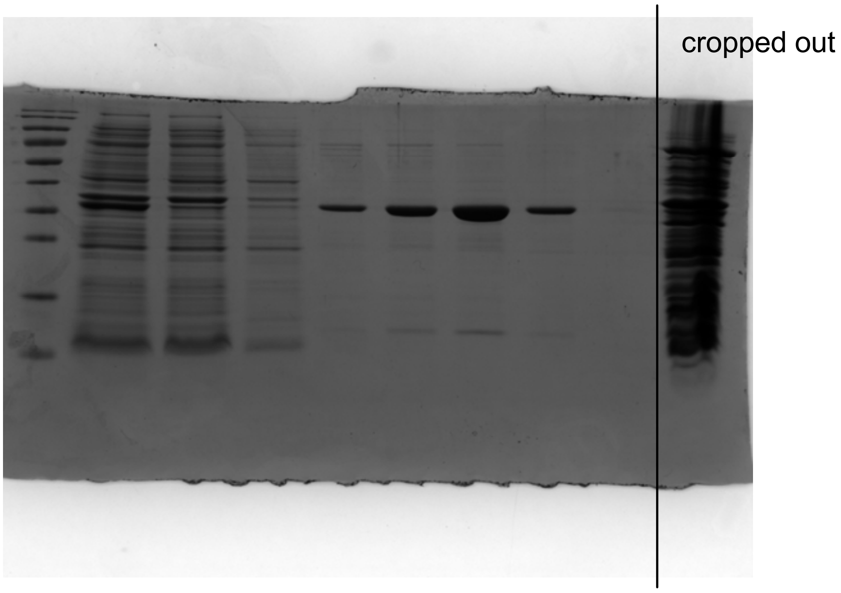

Full-length image of gel shown in Fig. 2a (bottom)

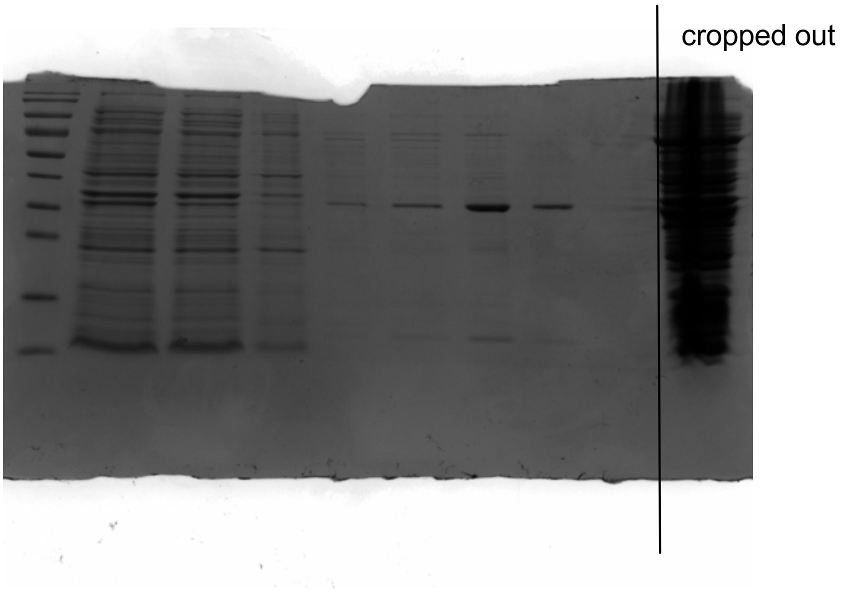

Full-length image of gel shown in Fig. 2b

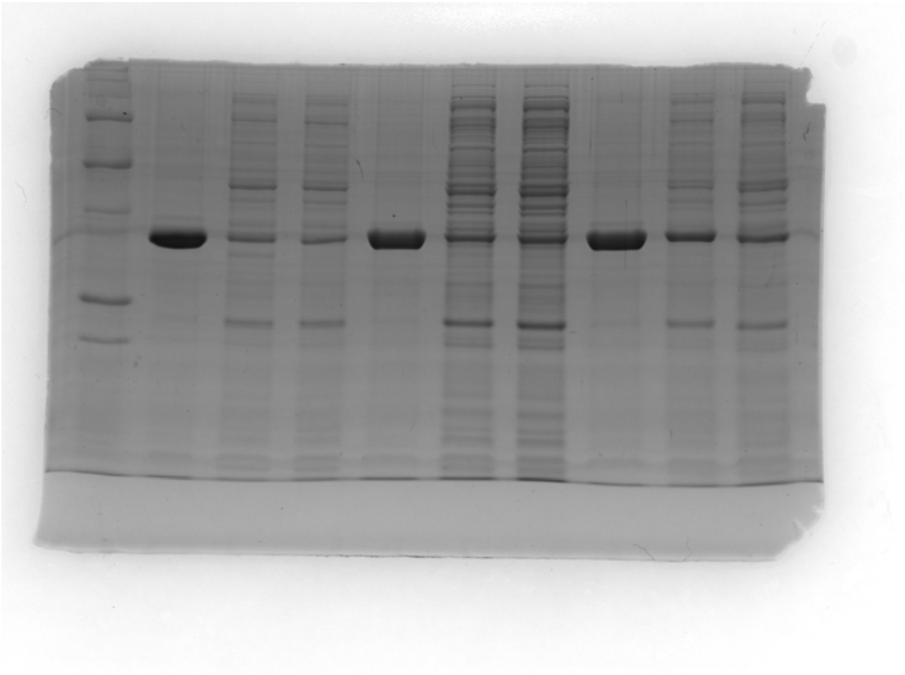

Full-length images of blot shown in Fig. 3a (multiple exposures)

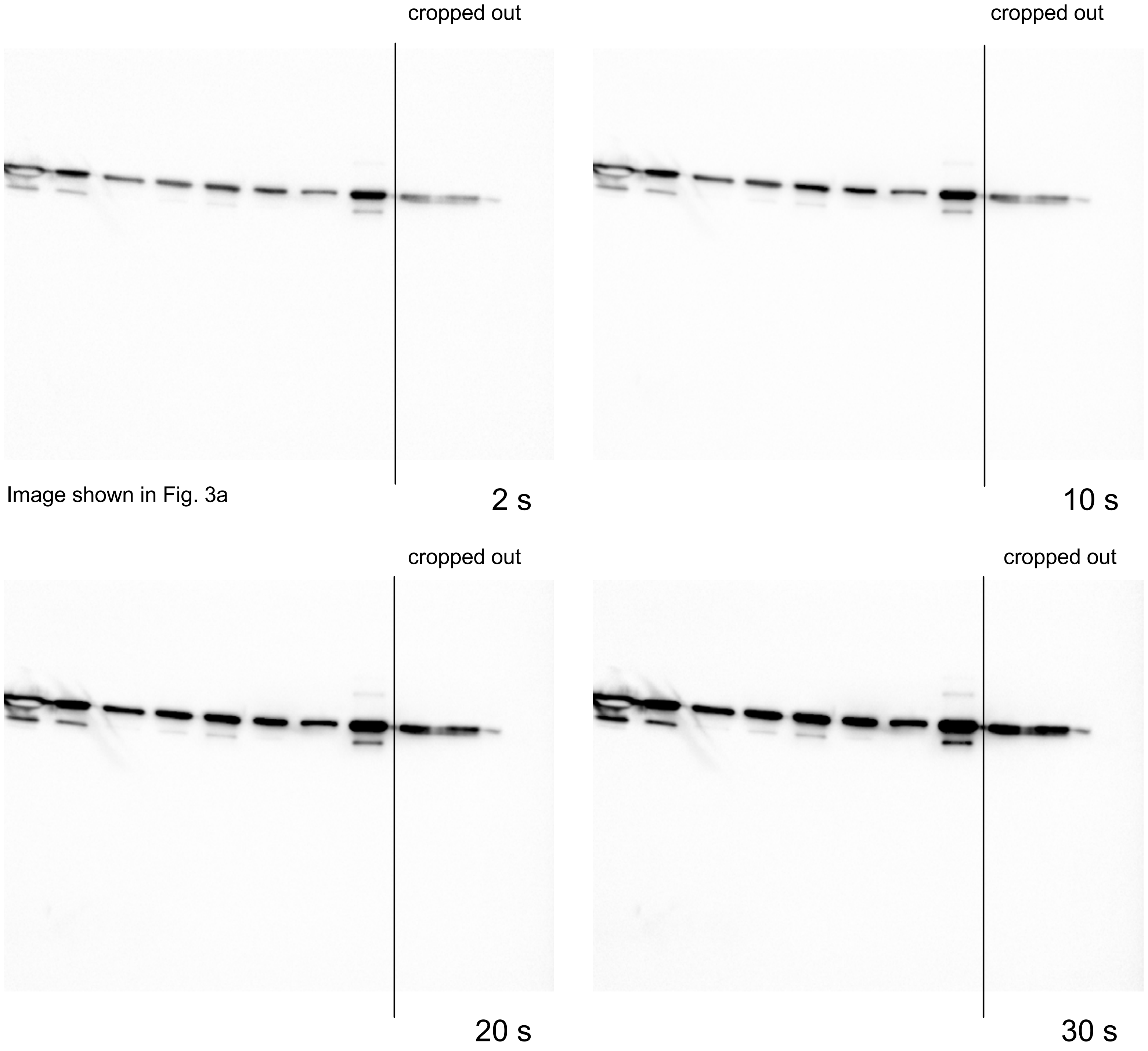

Full-length images of blot shown in Fig. 3b (multiple exposures)

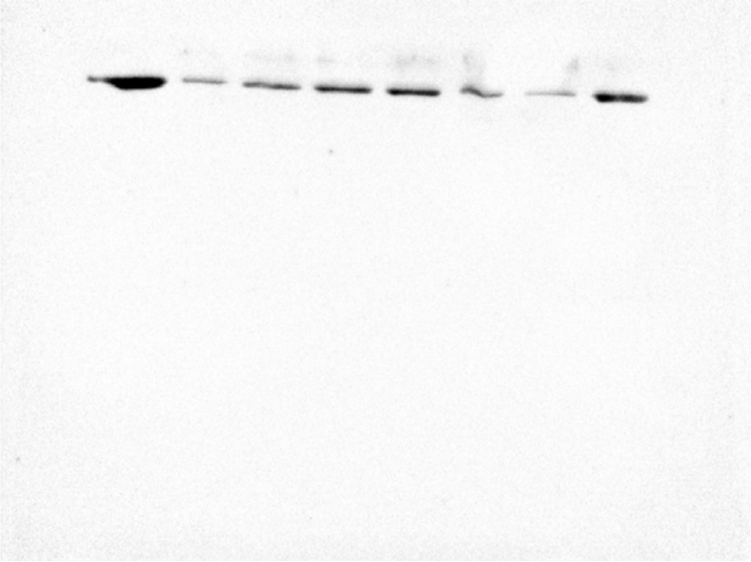

Image shown in Fig. 3b

26.1 s

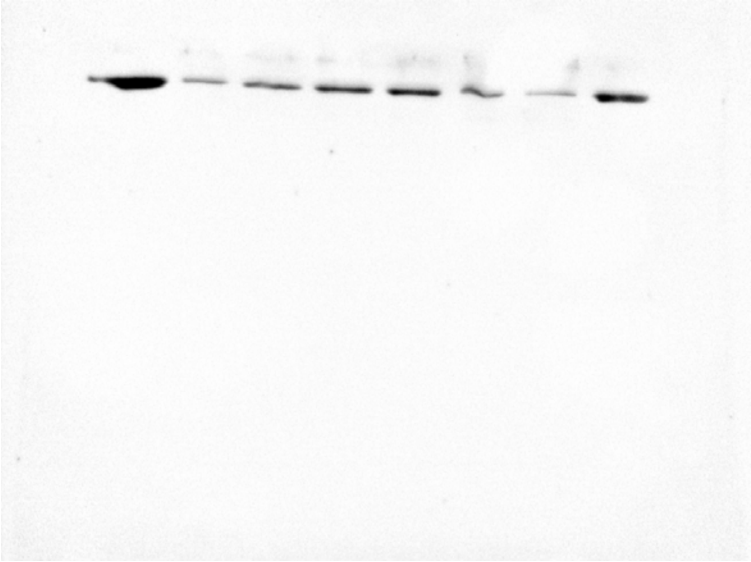

84.7 s

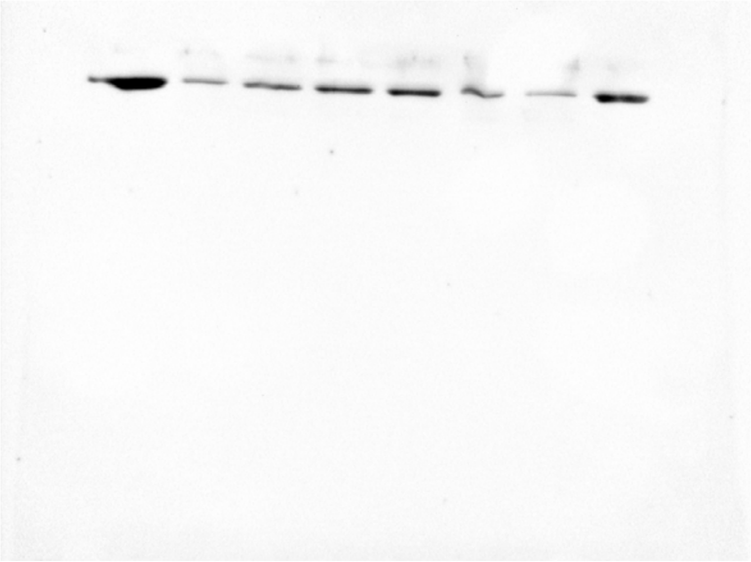

126.5 s

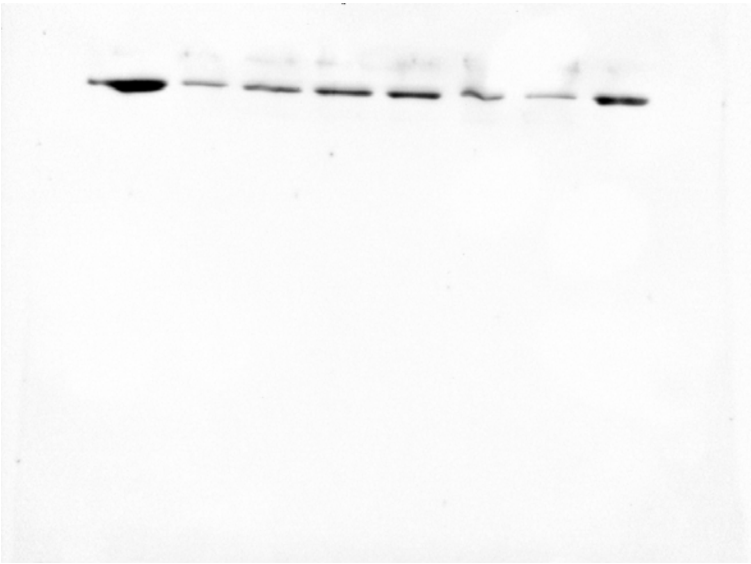

160 s

Full-length images of blot shown in Fig. 3c (multiple exposures)

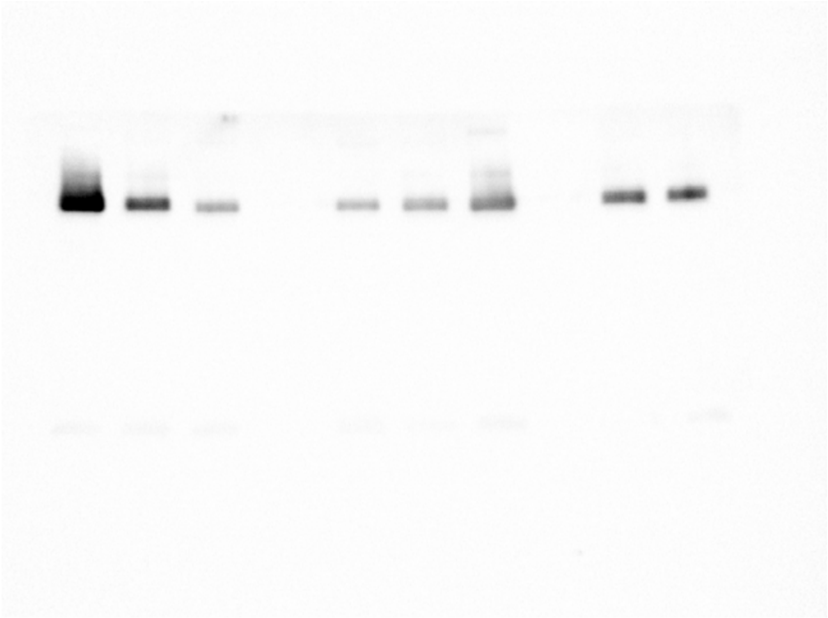

Image shown in Fig. 3c

3 s

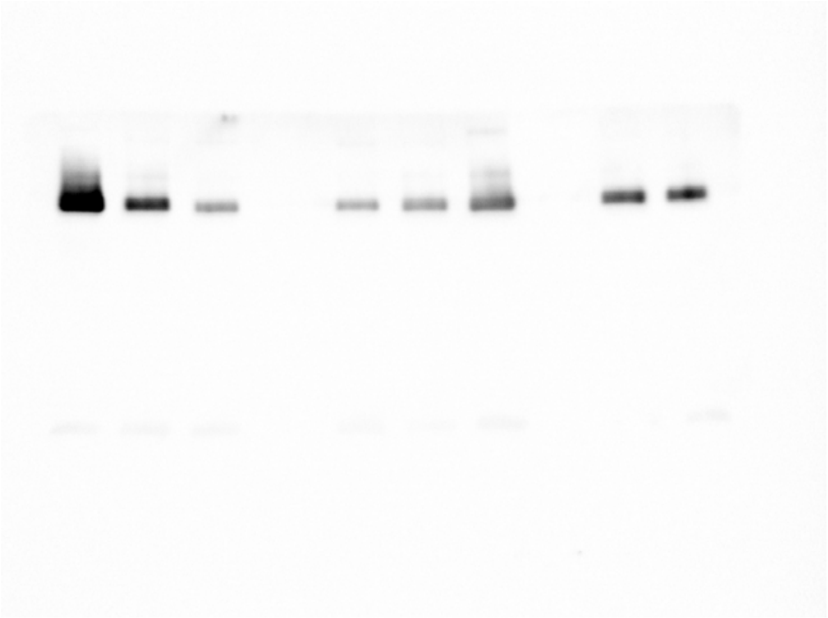

10 s

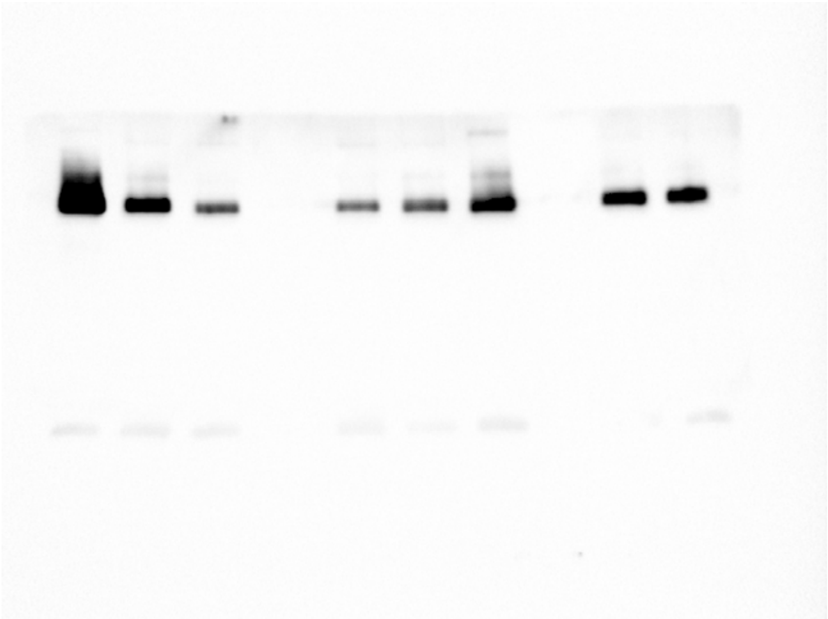

20 s

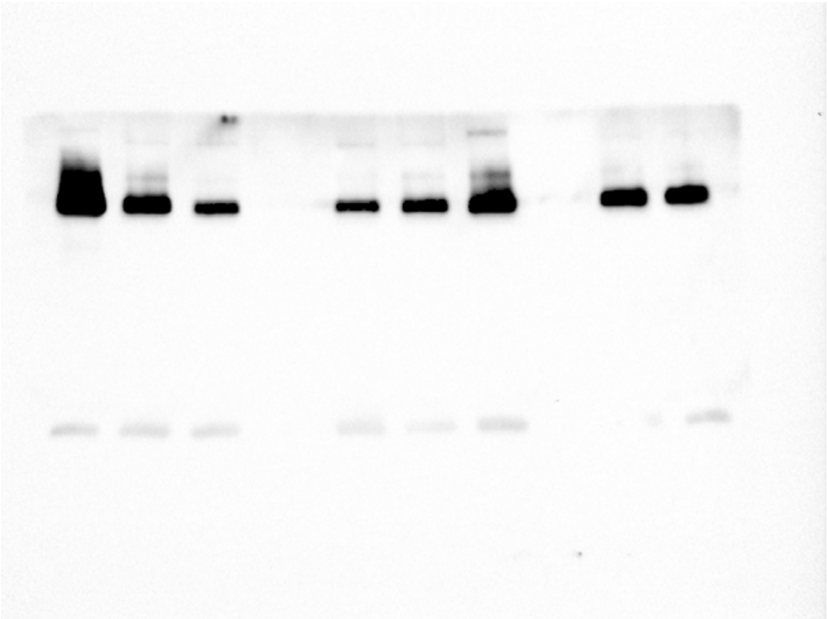

40 s

Full-length images of blot shown in Fig. 3d (multiple exposures)

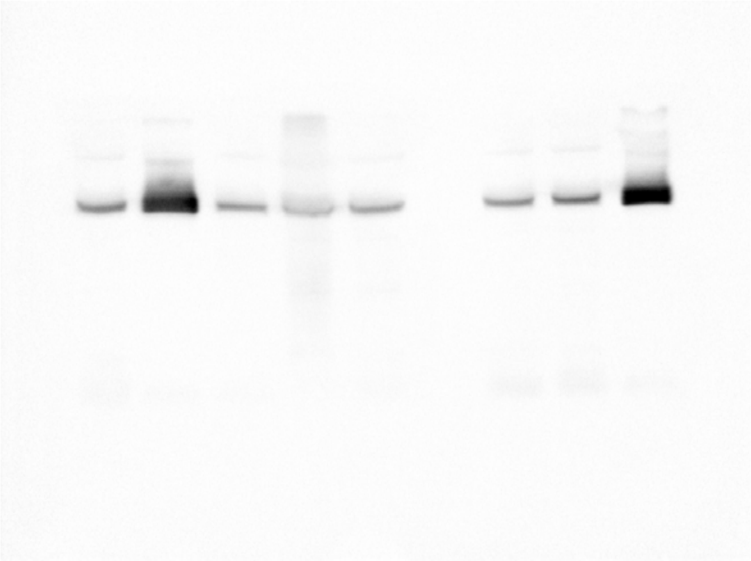

Image shown in Fig. 3d

15.2 s

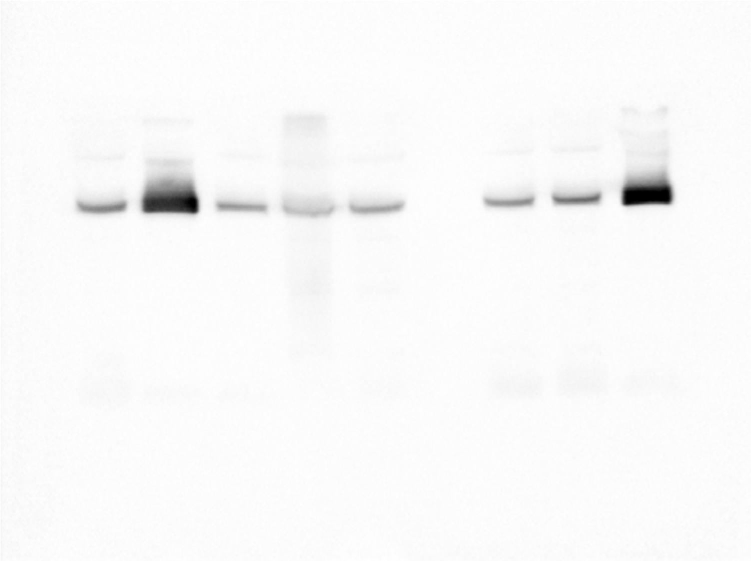

21.3 s

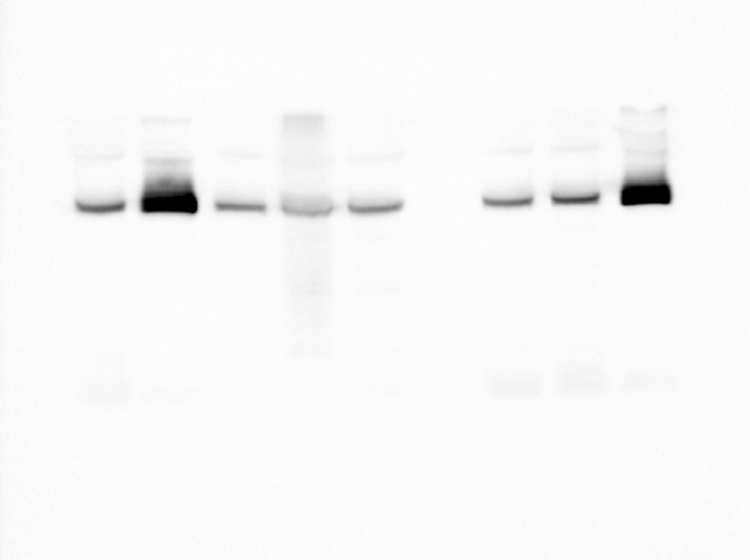

39.6 s

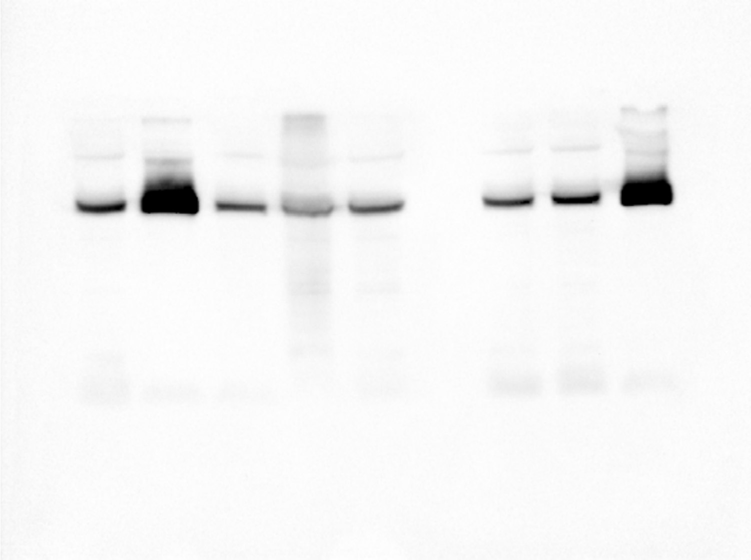

60 s

Full-length images of blot shown in Fig. S4a (multiple exposures)

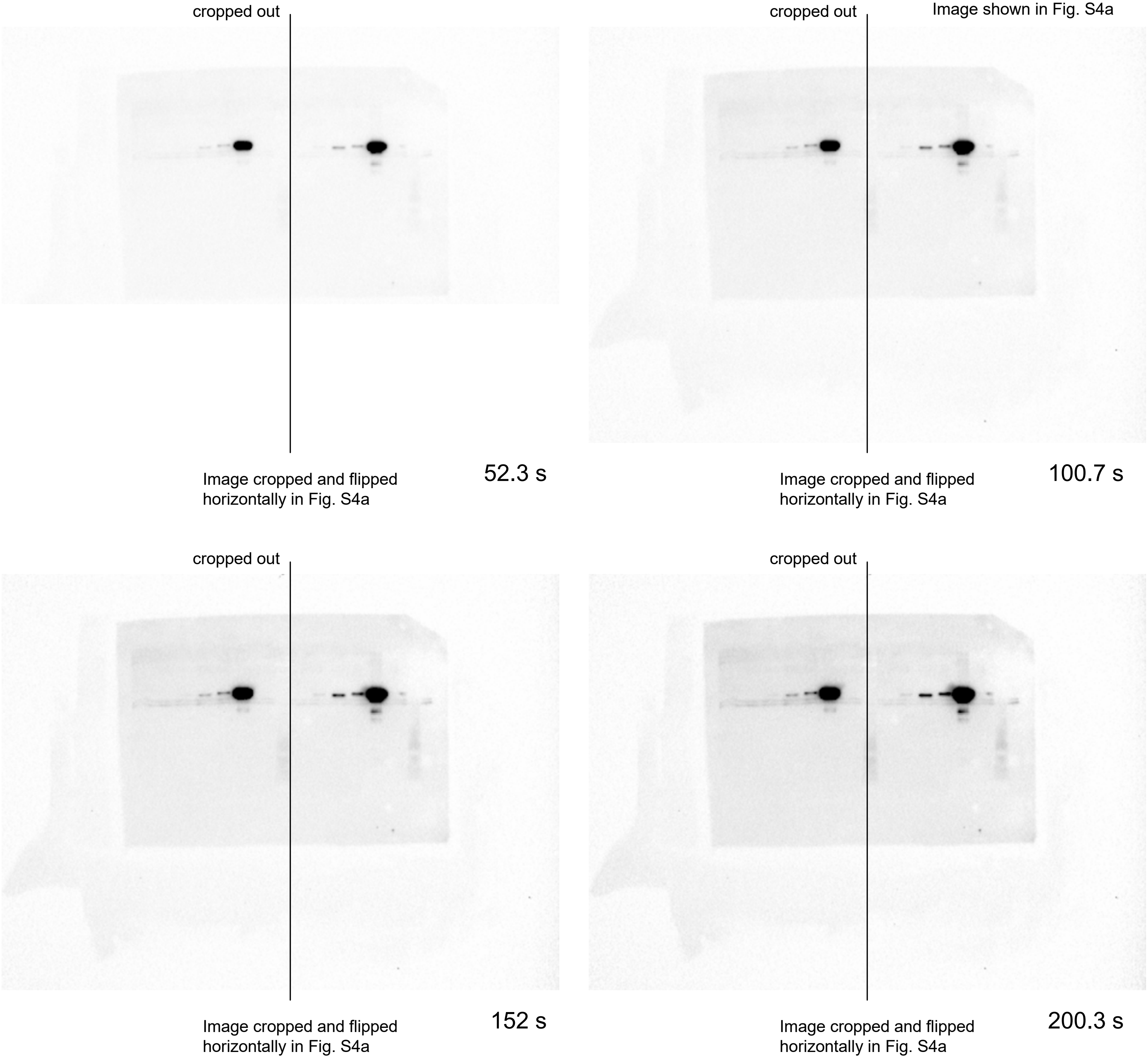

Full-length images of blot shown in Fig. S4b (multiple exposures)

cropped out

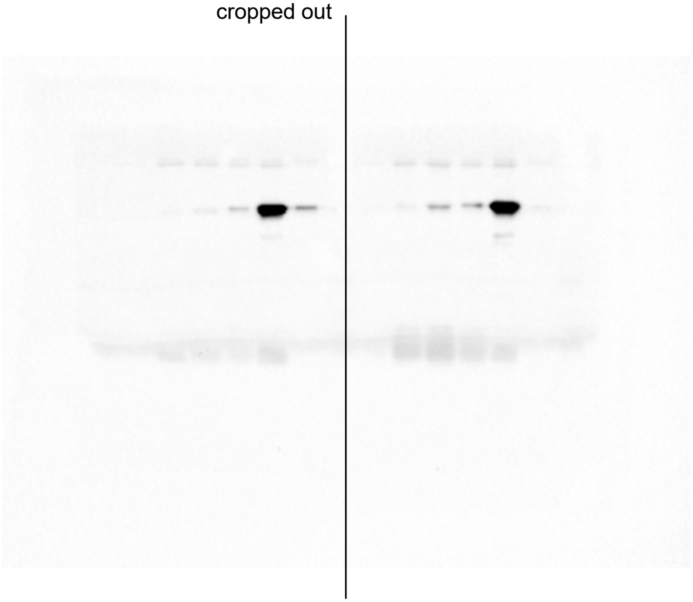

Image cropped and flipped horizontally in Fig. S4b

49.3 s

cropped out

Image shown in Fig. S4b

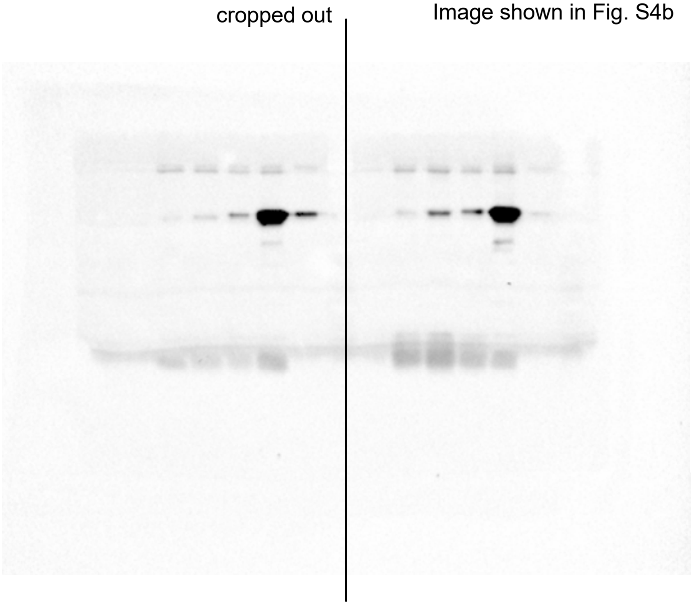

Image cropped and flipped horizontally in Fig. S4b

100.7 s

cropped out

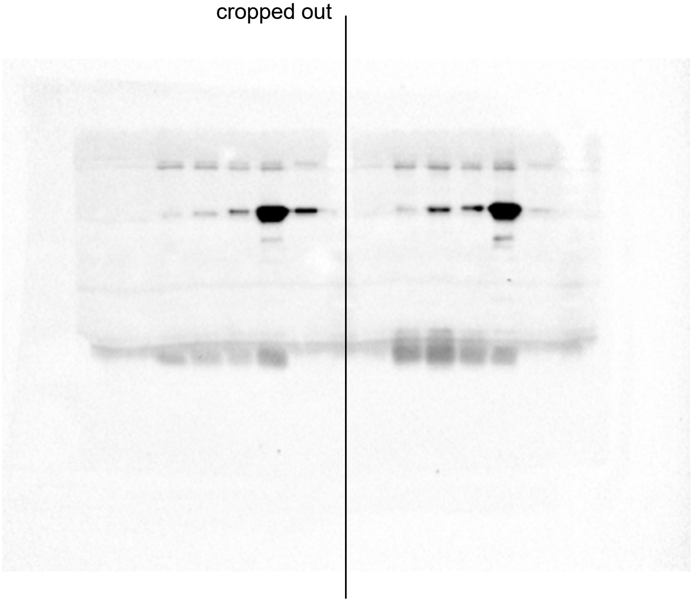

Image cropped and flipped horizontally in Fig. S4b

149 s

cropped out

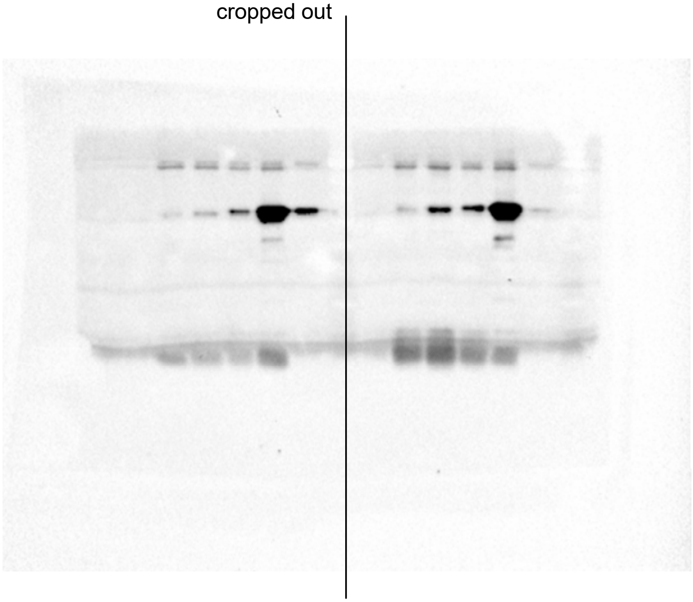

Image cropped and flipped horizontally in Fig. S4b

200.3 s
